# Ultrasound-triggered microbubble destruction enhances the radiosensitivity of glioblastoma by inhibiting PGRMC1-mediated autophagy in vitro and in vivo

**DOI:** 10.1186/s40779-022-00369-0

**Published:** 2022-02-14

**Authors:** Ying He, Xun-Hu Dong, Qiong Zhu, Ya-Li Xu, Ming-Liang Chen, Zheng Liu

**Affiliations:** 1 Department of Ultrasound, Xinqiao Hospital, Third Military Medical University (Army Medical University), Chongqing, 400037 China; 2 Department of Chemical Defense Medicine, School of Military Preventive Medicine, Third Military Medical University (Army Medical University), Chongqing, 400038 China; 3 Institute of Toxicology, School of Military Preventive Medicine, Third Military Medical University (Army Medical University), Chongqing, 400038 China; 4 Institute of Pathology and Southwest Cancer Centre, Southwest Hospital, Third Military Medical University (Army Medical University), Chongqing, 400038 China

**Keywords:** Ultrasound-triggered microbubble destruction, Radiosensitization, Progesterone receptor membrane component 1, Autophagy, Glioblastoma

## Abstract

**Background:**

Ultrasound-triggered microbubble destruction (UTMD) is a widely used noninvasive technology in both military and civilian medicine, which could enhance radiosensitivity of various tumors. However, little information is available regarding the effects of UTMD on radiotherapy for glioblastoma or the underlying mechanism. This study aimed to delineate the effect of UTMD on the radiosensitivity of glioblastoma and the potential involvement of autophagy.

**Methods:**

GL261, U251 cells and orthotopic glioblastoma-bearing mice were treated with ionizing radiation (IR) or IR plus UTMD. Autophagy was observed by confocal microscopy and transmission electron microscopy. Western blotting and immunofluorescence analysis were used to detect progesterone receptor membrane component 1 (PGRMC1), light chain 3 beta 2 (LC3B2) and sequestosome 1 (SQSTM1/p62) levels. Lentiviral vectors or siRNAs transfection, and fluorescent probes staining were used to explore the underlying mechanism.

**Results:**

UTMD enhanced the radiosensitivity of glioblastoma in vitro and in vivo (*P* < 0.01). UTMD inhibited autophagic flux by disrupting autophagosome-lysosome fusion without impairing lysosomal function or autophagosome synthesis in IR-treated glioblastoma cells. Suppression of autophagy by 3-methyladenine, bafilomycin A1 or *ATG5* siRNA had no significant effect on UTMD-induced radiosensitization in glioblastoma cells (*P* < 0.05). Similar results were found when autophagy was induced by rapamycin or ATG5 overexpression (*P* > 0.05). Furthermore, UTMD inhibited PGRMC1 expression and binding with LC3B2 in IR-exposed glioblastoma cells (*P* < 0.01). PGRMC1 inhibitor AG-205 or *PGRMC1* siRNA pretreatment enhanced UTMD-induced LC3B2 and p62 accumulation in IR-exposed glioblastoma cells, thereby promoting UTMD-mediated radiosensitization (*P* < 0.05). Moreover, PGRMC1 overexpression abolished UTMD-caused blockade of autophagic degradation, subsequently inhibiting UTMD-induced radiosensitization of glioblastoma cells. Finally, compared with IR plus UTMD group, PGRMC1 overexpression significantly increased tumor size [(3.8 ± 1.1) mm^2^ vs. (8.0 ± 1.9) mm^2^, *P* < 0.05] and decreased survival time [(67.2 ± 2.6) d vs. (40.0 ± 1.2) d, *P* = 0.0026] in glioblastoma-bearing mice.

**Conclusion:**

UTMD enhanced the radiosensitivity of glioblastoma partially by disrupting PGRMC1-mediated autophagy.

**Supplementary Information:**

The online version contains supplementary material available at 10.1186/s40779-022-00369-0.

## Background

Glioblastoma (WHO grade IV) is the most malignant primary intracranial neoplasm with poor prognosis [1]. At present, ionizing radiation (IR) is still one of the main treatment modalities for glioblastoma after maximum surgical resection [2]. Accumulating evidence has revealed that the weak response of glioma cells to IR leads to poor therapeutic outcomes, and the estimated median survival of glioblastoma patients is only 14.6 months [3]. Hence, the development of effective strategies to sensitize glioma cells to IR with fewer adverse effects is urgently needed. Ultrasound-triggered microbubble destruction (UTMD) is a new noninvasive method that can disrupt cell membrane integrity at a given site and induce increased capillary permeability through bursting of microbubbles (MBs), which has been widely used in the treatment of diverse diseases especially brain disorders in both military and civilian medicine [4]. Recently, UTMD has been promoted as a good candidate for enhancing the efficiency of radiotherapy and chemotherapy in various tumors [4–6]. It has been demonstrated that UTMD is a promising modality that could work synergistically with IR in liver, breast, bladder, and prostate tumor models [7]. Meanwhile, UTMD has been found to be an effective and safe method for unilaterally increasing blood–brain barrier permeability, thereby facilitating the release of drug-bearing nanoparticles in glioma, ultimately increasing their antitumor efficacy, indicating that UTMD would be an effective adjunct for glioblastoma treatment [8–11]. However, little information is available regarding the effects of UTMD on radiotherapy for glioblastoma as well as the underlying mechanism.

Autophagy is a highly conserved metabolic degradation process and is essential to maintain cellular homeostasis through the removal of damaged or redundant cell composition during stress conditions. In recent years, autophagy has been implicated in the pathogenesis and the outcomes of therapeutics in a wide spectrum of cancers including glioma [12]. Previous study has found that autophagy is highly activated in glioblastoma and blocking autophagy through genetic or pharmacological methods effectively inhibits the progression of glioma [13]. More recently, it has been demonstrated that inhibitors of dopamine receptor D4 or MTS4 selectively block the autophagic flux thereby leading to apoptosis and enhancing the antitumor effects of IR in glioma [14, 15]. It has also been found that downregulation of microRNA-93-mediated autophagy improves the response of glioblastoma to radiotherapy [16]. These results suggest that targeting autophagy might be an effective strategy to improve the efficacy of IR in glioblastoma. Furthermore, ultrasound (US) can promote mesenchymal stem cells (MSCs) chondrogenesis by inhibiting autophagy [17] and induce cell death by activating autophagy in nasopharyngeal carcinoma cells [18]. Autophagy inhibitors promote the chemosensitization effect of US in prostate cancer [19]. Simultaneously, UTMD induces autophagy and autophagy-related cell death in castration-resistant prostate cancer [20]. These data indicate the requirement of autophagy in the beneficial biological effects of UTMD, especially on cancer therapy.

Progesterone receptor membrane component 1 (PGRMC1) is a member of the membrane-associated progesterone receptor family that anchors to the cell membrane through the N-terminal transmembrane helix, and has been found to be highly expressed in multiple types of tumors, including glioma [21–23]. Accumulating evidence indicate that PGRMC1 is essential for tumor formation, invasion, and metastasis and is closely associated with an aggressive phenotype and poor prognosis of cancers [24]. Recently, researches have demonstrated that PGRMC1 mediates doxorubicin-induced chemoresistance in uterine sarcoma [4], and PGRMC1 knockdown increases the sensitivity of colon and liver cancers to erlotinib treatment [25], thus implicating PGRMC1 as a novel and promising target for cancer therapeutics. Moreover, the latest research has found that PGRMC1 induces autophagy through directly binding with light chain 3 beta 2 (LC3B2) [26], and hyperoside can sensitize ovarian cancer cells to cisplatin by activating PGRMC1-dependent autophagy [27]. These results support that PGRMC1-mediated autophagy might play a critical role in tumor progression and treatment. In addition, previous study has also confirmed that UTMD improves neuronal function in neurological diseases via modulating mechanoreceptors in the membranes [28], indicating that membrane receptors might play important roles in UTMD-mediated protective effects on neurological diseases. However, the effect of UTMD on PGRMC1 remains to be elucidated. Accordingly, this study aimed to investigate the effect of UTMD on the radiosensitivity of glioblastoma as well as the potential involvement of PGRMC1-mediated autophagy in vitro in cultured GL261, U251 cells and in vivo in orthotopic glioblastoma-bearing mice.

## Methods

### Antibodies and reagents

The green fluorescent protein (GFP)-LC3B plasmid was kindly provided by Dr. Tamotsu Yoshimori (Department of Cell Biology, National Institute for Basic Biology, Precursory Research for Embryonic Science and Technology, Okazaki, Japan). Dulbecco’s Modified Eagle’s Medium (DMEM, 11965167) and fetal bovine serum (26010074) were purchased from Gibco (USA). Cell counting kit-8 (CCK-8, CK04) was purchased from Dojindo Laboratories (Kumamoto, Japan). AG-205 (A1478) and an antibody against LC3B (L7543) were purchased from Sigma-Aldrich (St. Louis, MO, USA). Propidium iodide (PI, ST512), rapamycin (RAPA, S1842), and phosphate-buffered saline (PBS, C0221A) were purchased from the Beyotime Institute of Biotechnology (Haimen, China). 3-methyladenine (3-MA, HY-19312) and bafilomycin A1 (BafA1, HY-100558) were purchased from Med Chem Express (Monmouth Junction, NJ, USA). Antibodies against PGRMC1 (12990-1-AP) and ATG5 (10181-2-AP) were obtained from Proteintech (Wuhan, China). Antibodies against sequestosome 1 (SQSTM1/p62, ab56416/ab109012), Ki67 (ab15580), and lysosomal-associated membrane protein 1 (LAMP1, ab24170) were purchased from Abcam (Cambridge, UK), and an antibody against β-actin (ACTB, TA-09) was obtained from Zhongshan Jinqiao Biotechnology Co. (Beijing, China). An antibody against LAMP2 (DF6719) was got from Affinity Biosciences (Cincinnati, OH, USA). LysoTracker green fluorescent dye (LTG, L7526), LysoSensor green DND-189 (L7535), Premo™ Autophagy Tandem Sensor Red fluorescent protein (RFP)-GFP-LC3B (P36239), and Lipofectamine™ RNAiMAX transfection reagent (13778150) were purchased from Invitrogen (Carlsbad, CA, USA).

### Preparation of MBs

MB ampoules (ZHIFUXIAN) were newly manufactured by us as described previously [29]. Briefly, dipalmitoyl phosphatidylglycerol, distearoyl phosphatidylcholine, and polyethylene glycol-4000 were mixed with ultrapure water at a mass ratio of 30:30:3000 (W:W) at 60 °C in a thermostatic water bath. Thereafter, the resulting suspension was dispersed into 2 ml vials (1 ml/vial), which were lyophilized with a freeze drier. The powder prepared by lyophilization was dispersed in 1 ml of a mixed solution of 50% glucose, propylene glycol and glycerin with a volume ratio of 8:1:1, and the air in the vial was substituted with perfluoropropane. The vial was closed tightly and oscillated with a mechanical shaker (AT & M, Beijing, China) with a frequency of 4000–5500/min for 60 s to form the ZHIFUXIAN MBs, which had a concentration and mean diameter of (4–9) × 10^9^/ml and 2.13 μm, respectively.

### UTMD parameters in vitro

In the current study, UTMD was performed as described before with some modifications [30]. As shown in Additional file 1: Fig. S1a, the transducer of metron accusonic US machine AP-170 (Metron, Australia) was used as a MBs trigger in this study. GL261 and U251 cells were treated at ultrasonic intensities of 0.4, 0.8, 1.2 and 2.0 W/cm^2^ with different concentrations of MBs (50, 100, 200, and 400 μl/ml) at a duty cycle of 10% for 60 s.

### Cell culture and treatments

Murine GL261 and human U251 glioblastoma cells were purchased from Fuheng Biology (Shanghai, China) and cultured in DMEM supplemented with 10% fetal bovine serum at 37 °C in a humidified atmosphere containing 5% CO_2_. All experiments were performed following 3–6 passages when the cells reached 80–90% confluence.

As shown in Additional file 1: Fig. S1a, during the logarithmic growth phase, GL261 and U251 cells were divided into the following groups: 1) control; 2) IR; 3) IR + UTMD. In IR group, cells were treated by a single dose of IR (2 Gy) using an RS-2000 irradiator (Rad Source Technologies, Atlanta, GA, USA) at a dose rate of 1 Gy/min with 160 kVp X-rays using a 0.3-mm copper filter. In IR + UTMD group, cells were pretreated with UTMD immediately followed by a single dose of IR (2 Gy). All treatments were carried out in complete culture medium to prevent the induction of autophagy through the serum starvation pathway. In order to determine the exact role of autophagy in UTMD-induced radiosensitization of glioblastoma cells, the following groups were set up: 1) control; 2) IR; 3) IR + UTMD; 4) IR + UTMD + 3-MA; 5) IR + UTMD + BafA1; 6) IR + UTMD + RAPA; 7) IR + RAPA; 8) IR + 3-MA; 9) IR + BafA1. IR and IR + UTMD groups were treated as described above. 3-MA, a selective PI3K inhibitor, was used to block autophagosome formation in IR + UTMD + 3-MA and IR + 3-MA groups; BafA1, which inhibits the acidification of organelles and, subsequently, autophagosome-lysosome fusion, was chosen to disrupt autophagic flux in IR + UTMD + BafA1 and IR + BafA1 groups; RAPA, a selective inhibitor of mammalian target of rapamycin (mTOR) was used to induce autophagy in IR + UTMD + RAPA and IR + RAPA groups. Meanwhile, AG-205, a selective inhibitor of PGRMC1 was also applied to investigate the role of PGRMC1 in UTMD-induced radiosensitization of glioblastoma cells. Where indicated, cells were treated with 3-MA (5 mmol/L), BafA1 (10 nmol/L), AG-205 (10 µmol/L), or RAPA (20 nmol/L) for 1 h followed by IR or IR plus UTMD treatment for another 24 h.

### Animals treatments

Female C57BL/6J mice (*n* = 120) approximately 6-week-old weighing 14–20 g were purchased from Hunan SJA Laboratory Animal Co. Ltd. (Hunan, China) and maintained at a controlled temperature (22 ± 2) °C with a 12-h light/dark cycle and ad libitum access to water and food. All animal experiments were approved by the Animal Care and Use Committee of the Army Medical University (AMUWEC2020304, Chongqing, China).

The schedule of animal treatment was indicated in Additional file 1: Fig. S1b. Six-week-old female C57BL/6J mice (*n* = 120) were inoculated in the right caudate putamen with GL261 cells to establish the orthotopic glioblastoma mouse model as described before [10]. Briefly, for the implantation of GL261 cells, a sagittal incision was made through the skin overlying the calvarium, and a small dental drill was used to create a hole (1.0 mm diameter) in the 1 mm anterior, 2.5 mm lateral to the bregma (right hemisphere of the brain) without duramatral damage. GL261 cells expressing luciferase were transduced with shPGRMC1-expressing vector or control vector, and then a total of 1 × 10^5^ GL261 cells were injected at a depth of 3.5 mm within a 10 min period. Seven days after tumor implantation, the tumor size was evaluated by in vivo bioluminescent imaging using an IVIS Spectrum system (PerkinElmer, Waltham, MA, USA), and data were quantified by Living Image Software (PerkinElmer). Then, the mice were randomly allocated to the following three treatment groups (*n* = 12 each group): 1) control; 2) IR; 3) IR plus UTMD.

Before the experiments, pentobarbital sodium anesthetic (50 mg/kg) was intraperitoneally injected to anesthetize mice. Then, MBs (1% or 3% V/V per mouse) were continuously intravenously administered via the tail vein, and US transmission gel was coupled on the surface of the mouse brain corresponding to the glioma in IR plus UTMD groups. The same volume of normal saline was continuously intravenously administered via the tail vein in control and IR groups. A linear array probe (X4-12L) of a VINNO 70 diagnostic US scanner (Vinno Technology Co. Ltd., Suzhou, China) was applied to generate the UTMD effect, and the transducer was placed over the glioma tumor. Mice were subjected to US for 5 min (3 MHz frequency, 50 Hz pulse repetition frequency and 26 cycles pulse length). The MI value was approximately 0.4 or 0.8, the transmitting/intermittent time was 0.62/2 s, and the depth was set at 1 cm. Immediately following UTMD treatment, the animals received 2 Gy whole-brain irradiation using an RS-2000 irradiator (Rad Source Technologies). Combined treatment with UTMD (every other day) and IR (every day) was administered over a period of 5 d (10 Gy total) in IR plus UTMD group. In IR group, mice were only treated with IR (every day) over a period of 5 d (10 Gy total). On day 20 after tumor transplantation, US and bioluminescent imaging were used to determine the tumor size. Meanwhile, mice were euthanized, and brain tumors were collected. Ki67 levels were determined by immunofluorescence, and the expression of PGRMC1, p62 and LC3B2 were monitored by Western blotting. Survival was also investigated in the different treatment groups.

### Cell viability measurement

The CCK-8 detection kit was used to measure cell viability as previously described [31]. Briefly, GL261 and U251 cells were seeded in a 96-well microplate (3650, Corning Life Sciences, USA) at a density of 5000 cells/well and then subjected to IR at a series of doses (2, 4, 8 and 10 Gy). After 24 h, CCK-8 solution (20 μl/well) was added to the wells, and the plate was incubated at 37 °C for 2 h. Viable cells were assessed according to absorbance measurements at a wavelength of 450 nm with a Monochromator Microplate Reader (Safire II; Tecan Group Ltd., Männedorf, Switzerland). The percent cell viability was reported as the optical density value at 450 nm of the treatment group relative to that of the control group (set as 100%).

### Cell death assay

PI staining was used to assess cell death as previously reported [31]. Briefly, the cells were trypsinized with 0.5 ml of 0.25% trypsin for 1 min, collected, and resuspended in 1 ml of PBS. The cells were then incubated with 0.5 ml of staining solution (10 mg/ml PI) at 37 °C for 30 min in the dark. Cell death was assessed by Flow Cytometry (BD FAC Scan Flow cytometer, BD Bioscience, USA).

### Clonogenic assay

For the colony formation assay, 800 GL261 cells and 1000 U251 cells were plated into each well of 6-well plates, respectively. Twenty-four hours later, glioblastoma cells were treated as indicated. At the end of treatment, the media were totally drawn off and fresh media were inserted. Cells were then incubated for 10 d for GL261 cells or 14 d for U251 cells. Colonies were fixed with 4% paraformaldehyde and stained with 0.1% crystal violet (C0121, Beyotime Biotechnology, China). The colonies were counted using Image-Pro Plus 6.0 software (MEDIA Cybernetics, USA).

### Western blotting analysis

Cells and brain tumors were collected, lysed and subjected to Western blotting as described previously [31]. Briefly, 20–150 µg of protein was resolved by 10–15% SDS-PAGE and then electroblotted onto polyvinylidene difluoride membranes for Western blotting analysis. Blots were probed with 1:500–1:1000-diluted primary antibodies overnight at 4 °C, followed by horseradish peroxidase-conjugated secondary antibodies (31340 and 31455, Thermo Scientific Lab Vision, USA). The protein bands were visualized using an enhanced chemiluminescence system, and densitometric analysis was performed using Scion Image-Release Beta 4.02 software (http://scion-corporation.software.informer.com*).*

### Measurement of GFP-LC3B dots

GL261 cells were transfected with plasmids expressing GFP-LC3B as previously reported [31]. After 24 h, the cells were exposed to various indicated treatments. Then, the cells were washed with PBS, fixed by incubation for 20 min at 37 °C in 4% paraformaldehyde, permeabilized with 0.1% (V/V) Triton X-100, and washed with PBS containing 2% fetal bovine serum albumin. All steps were performed at room temperature. A ZEISS LSM900 Confocal Laser Scanning Microscope (ZEISS, Germany) was used for confocal microscopy analysis and was performed under the same microscope settings including the exposure intensity of laser, pinhole, master gain, digital offset, and digital gain to guarantee that the results from different groups were comparable, respectively.

### Transmission electron microscopy

GL261 cells were collected and fixed in 2% paraformaldehyde and 0.1% glutaraldehyde in 0.1 mol/L sodium cacodylate for 2 h, postfixed with 1% OsO_4_ for 1.5 h, washed, and stained for 1 h in 3% aqueous uranyl acetate. The samples were then washed again, dehydrated with graded alcohol, and embedded in Epon-Araldite resin (034, Canemco & Marivac, Canada). Ultrathin sections were cut with an ultramicrotome (Reichert-Jung, Inc., Cambridge, UK), counterstained with 0.3% lead citrate, and examined on a transmission electron microscope (model No. EM420, Koninklijke Philips Electronics N.V., Amsterdam, The Netherlands).

### RFP-GFP-LC3 assay

The Premo™ Autophagy Tandem Sensor RFP-GFP-LC3B, which is a lentivirus carrying expression cassettes that encode tandem fluorescence-tagged LC3B, was used to evaluate the number of autophagosomes and autolysosomes following the manufacturer’s instructions. Briefly, 1 × 10^5^ GL261 cells were grown on glass-bottom dishes and infected with lentivirus for 24 h. Then, the GL261 cells were treated with or without UTMD (ultrasonic intensity at 1.2 W/cm^2^ with 200 μl/ml MBs at a duty cycle of 10% for 60 s) following IR (2 Gy). After 24 h, all samples were examined by a ZEISS LSM900 Confocal Laser Scanning Microscope equipped with a 40 × objective and were performed under the same microscope settings including the exposure intensity of laser, pinhole, master gain, digital offset, and digital gain to guarantee that the results from different groups were comparable, respectively.

### siRNA assay

siRNAs for *ATG5* (sc-41446) and *PGRMC1* (sc-76112) were purchased from Santa Cruz Biotechnology along with control siRNA (sc-44230). Lipofectamine RNAiMAX was diluted in Opti-MEM® I reduced serum medium (31985070, Gibco) according to the manufacturer’s protocol. Glioblastoma cells were transfected with 80 nmol/L siRNA with 4 μl Lipofectamine RNAiMAX for 5–7 h according to the manufacturer’s protocol. Then, the cells were switched to complete DMEM and incubated for an additional 24 h. Where indicated, the cells were treated with or without UTMD (ultrasonic intensity at 1.2 W/cm^2^ with 200 μl/ml MBs at a duty cycle of 10% for 60 s) following IR (2 Gy). After 24 h, the cells were harvested, and Western blotting analysis was performed.

### Lentiviral construction and cell transfection

The lentiviral vectors encoding the mouse *ATG5* (46467-2) or *PGRMC1* (64528-1) gene were purchased from Gene Chem (Shanghai, China). The processes of transduction and the establishment of stable cell lines were executed according to the manufacturer's instructions.

### LysoTracker green fluorescent dye and LysoSensor green DND-189 staining

Cells were cultured overnight on glass-bottom dishes at a density of 8000 cells/dish or in 96-well microplates at a density of 5000 cells/well and then exposed to the various indicated treatments. Thereafter, the cells were washed twice with fresh medium and loaded with LysoTracker green fluorescent dye (LTG, which stains acidic compartments, particularly lysosomes, 50 nmol/L) or LysoSensor green DND-189 (which can exhibit a pH-dependent increase in fluorescence intensity upon acidification, 1 μmol/L) for 10–15 min in humidified air at 37 °C in DMEM culture medium without fetal bovine serum. Afterward, the cells were washed three times with PBS, and fluorescence intensity was measured by a ZEISS LSM900 Confocal Laser Scanning Microscope under the same microscope settings including the exposure intensity of laser, pinhole, master gain, digital offset, and digital gain to guarantee that the results from different groups were comparable, respectively or was quantified using an Infinite™ M200 Microplate Reader (Tecan Group Ltd.).

### Detection of cathepsin B and cathepsin D activity

The catalytic activity of cathepsin B (CTSB) and cathepsin D (CTSD) was determined with CTSB (K140-100, BioVision, USA) and CTSD (K143-100, BioVision) activity fluorometric assay kits in accordance with the manufacturer’s instructions. The protein concentrations were assessed with a BCA protein assay kit (Beyotime Biotechnology). The samples were assessed using an Infinite™ M200 Microplate Reader (Tecan Group Ltd.)

### Immunofluorescence analysis

Cells were seeded in 15-mm confocal dishes and treated as indicated. Then, the cells were fixed with 4% paraformaldehyde for 10 min at room temperature. On day 20 after tumor explantation, the brain tumors were collected and embedded in OCT at − 20 °C. Then, the tissues were sectioned at a thickness of 6–8 μm. For immunofluorescence analysis, the cells and sections were washed three times with PBS for 5 min, permeabilized with 0.3% (V/V) Triton X-100 for 10 min and blocked in 3% BSA for 1 h at room temperature. Thereafter, the sections were incubated with rabbit anti-LC3B antibody (1:300), mouse anti-PGRMC1 (1:200) and mouse anti-Ki67 (1:100) overnight at 4 °C. Then, the sections were incubated with the appropriate Alexa Fluor 594 goat anti-rabbit IgG (H + L) antibody (A11012, Invitrogen) or Alexa Fluor® 488 goat anti-mouse IgG (H + L) antibody (A11029, Invitrogen) for 2 h at room temperature. DAPI staining solution (C1005, Beyotime Biotechnology) was used to stain the nuclei for 10 min at room temperature. Finally, the sections were mounted on glass slide. All digital images of the dishes and sections were acquired using a ZEISS LSM900 Confocal Laser Scanning Microscope under the same microscope settings including the exposure intensity of laser, pinhole, master gain, digital offset, and digital gain to guarantee that the results from different groups were comparable, respectively.

### US detection of orthotopic glioblastoma

A high-frequency US system (Vevo 2100, VisualSonics, Toronto, Canada) equipped with a linear array transducer (MS-400, 30 MHz center frequency) was used to detect orthotopic glioblastoma as described previously [32]. Briefly, glioblastoma-bearing mice were anesthetized by intraperitoneal injection of 50 mg/kg pentobarbital sodium (1% in normal saline). The fur at the imaging location was shaved, and warm ultrasound gel was liberally applied to ensure optimal image quality. A 13-mm width and 12-mm scanning depth field of view were used. Imaging was performed with the animal in the prone position with the transducer in the coronal plane starting at the vortex and moving caudally in a slow, controlled manner. Sagittal imaging was performed perpendicular to the coronal imaging starting at the midline and moving laterally on both sides. The machine setting was optimized to obtain a 50-μm resolution with frame rates in 2D up to 628 frames per second for a 4 mm × 4 mm field of view, as recommended by the manufacturer (VisualSonics). To reduce variability, the image parameters remained constant throughout the experiment. All examinations were performed by an experienced operator, and all measurements were repeated three times at the same site.

### Statistical analysis

All statistical analysis was performed in SPSS 18.0 statistical software (SPSS Inc., Chicago, IL, USA). Quantitative data are presented as the mean ± standard deviation (SD) of three experiments. Significance between individual group means was computed using a Student’s *t*-test, while variations between several group means were determined using a one-way analysis of variance. Survival analyses were carried out using the Kaplan–Meier method with the log-rank test used for comparison. Differences with a *P*-value < 0.05 were considered statistically significant.

## Results

### UTMD enhanced the radiosensitivity of glioblastoma cells

**Fig. 1:**
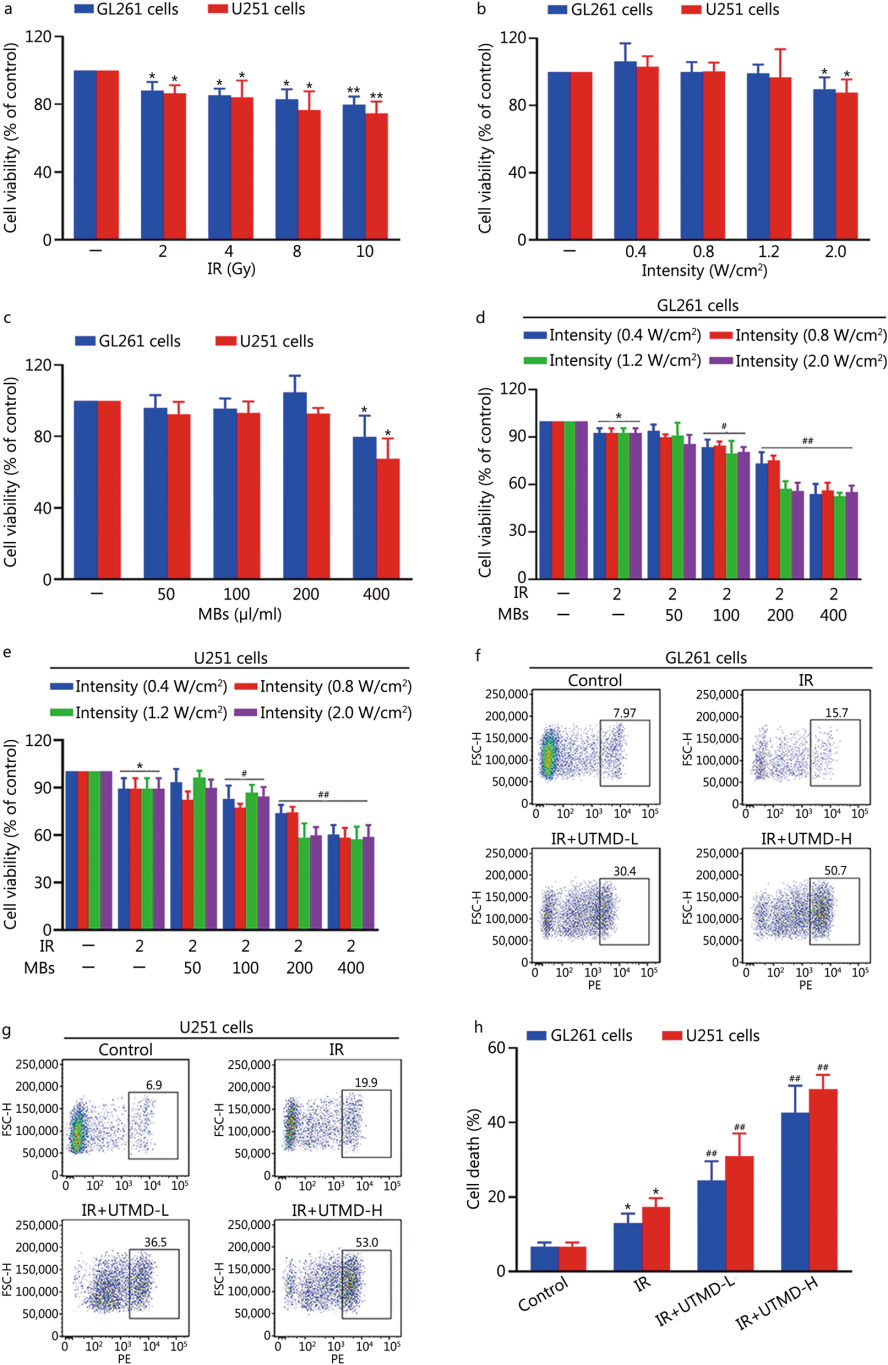
UTMD enhanced the radiosensitivity of glioblastoma cells. **a** GL261 and U251 cells were exposed to different doses of IR (2, 4, 8, and 10 Gy), and after 24 h, cell viability was measured using a CCK-8 detection kit. **b** Cells were treated at ultrasonic intensities of 0.4, 0.8, 1.2 and 2.0 W/cm^2^ at a duty cycle of 10% for 60 s and then incubated for another 24 h, and the cell viability of different experimental groups was detected using the CCK-8 detection kit. **c** Cells were treated with MBs (50, 100, 200, and 400 μl/ml) at an intensity of 1.2 W/cm^2^ and a duty cycle of 10% for 60 s. Twenty-four hours later, cell viability was measured using a CCK-8 detection kit. **d**, **e** GL261 and U251 cells were treated at ultrasonic intensities of 0.4, 0.8, 1.2 and 2.0 W/cm^2^ with different concentrations of MBs (50, 100, 200, and 400 μl/ml) at a duty cycle of 10% for 60 s. Then, the cells were exposed to IR (2 Gy). After 24 h, the cell viability of different experimental groups was detected using the CCK-8 detection kit. **f**, **g** GL261 and U251 cells were treated with UTMD-L (ultrasonic intensity of 1.2 W/cm^2^ combined with 100 μl/ml MBs) or UTMD-H (ultrasonic intensity of 1.2 W/cm^2^ combined with 200 μl/ml MBs) at a duty cycle of 10% for 60 s, and then the cells were exposed to IR (2 Gy). After 24 h, cell death was determined by PI staining followed by flow cytometry. **h** The quantifications of cell death. Values are presented as mean ± SD (*n* = 3). *P < 0.05, **P < 0.01 vs. the vehicle-treated control group; ^#^*P* < 0.05, ^##^*P* < 0.01 vs. IR group. UTMD ultrasound-triggered microbubble destruction, IR ionizing radiation, MBs microbubbles, CCK-8 cell counting kit-8, PI propidium iodide, SD standard deviation

We found that glioblastoma cells were resistant to IR exposure, 2 Gy IR induced a slight but significant decrease in viability of approximately (10.8 ± 5.0)% in GL261 cells or (13.5 ± 4.7)% in U251 cells as well as inhibiting colony formation of glioblastoma cells, respectively (*P* < 0.05, Fig. 1a and Additional file 1: Fig. S2). Accordingly, IR at a single dose of 2 Gy was used in the following experiments to identify the effect of UTMD on the radiation response of glioblastoma cells.

As shown in Fig. 1b, an ultrasonic intensity of up to 1.2 W/cm^2^ was tolerated by glioblastoma cells. When the ultrasonic intensity was fixed at 1.2 W/cm^2^, the addition of MBs at concentrations of up to 200 μl/ml was tolerated by glioblastoma cells (Fig. 1c). Moreover, when the MBs concentration was ≥ 100 μl/ml, UTMD notably enhanced the response to IR at different ultrasonic intensities in glioblastoma cells (Fig. 1d, e). In GL261 cells, the cell viability of IR plus UTMD (ultrasonic intensity of 1.2 W/cm^2^ with 200 μl/ml MBs) group was (57.3 ± 4.8)%, which was similar to that of the IR plus UTMD (ultrasonic intensity of 2.0 W/cm^2^ with 200 μl/ml MBs) group [(56.0 ± 5.3)%] and the IR plus UTMD (ultrasonic intensity of 1.2 W/cm^2^ with 400 μl/ml MBs) group [(55.2 ± 4.1)%]. Similar results were found in U251 cells (Fig. 1d, e).

Meanwhile, with PI staining, we also found that UTMD-L (ultrasonic intensity of 1.2 W/cm^2^ combined with 100 μl/ml MBs) and UTMD-H (ultrasonic intensity of 1.2 W/cm^2^ combined with 200 μl/ml MBs) conditions markedly increased cell death in IR-treated glioblastoma cells (Fig. 1f–h). These results suggested that UTMD improved the IR response of glioblastoma cells. Accordingly, ultrasonic intensity of 1.2 W/cm^2^ combined with 200 μl/ml MBs was set as the optimal parameter for treating cells in subsequent experiments to clarify the underlying mechanism of UTMD-induced enhancement of IR response in glioblastoma cells.

### UTMD inhibited autophagy in IR-treated glioblastoma cells

**Fig. 2:**
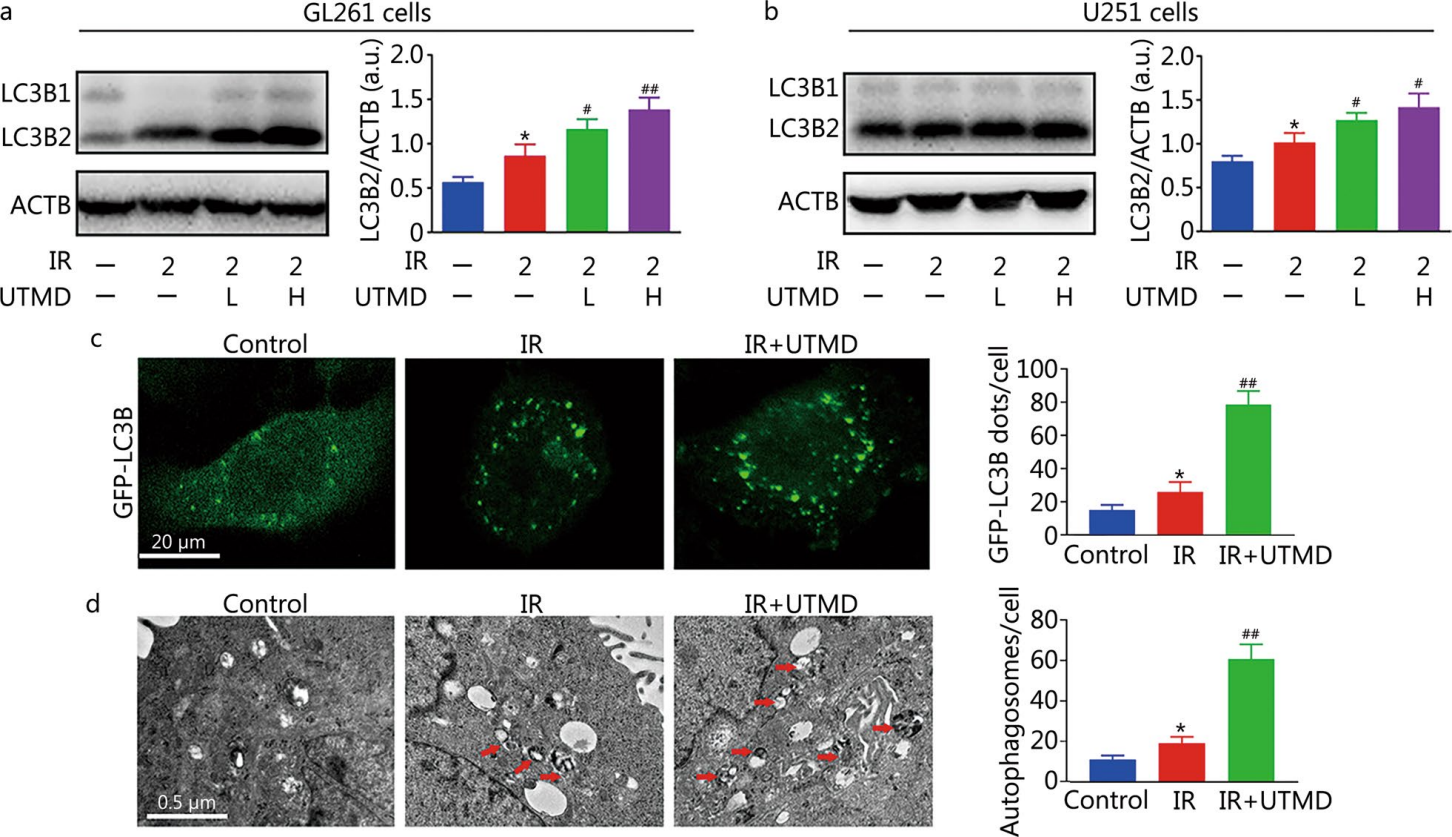
UTMD-induced autophagosome accumulation in IR-exposed glioblastoma cells. **a**, **b** GL261 and U251 cells were treated with UTMD-L (ultrasonic intensity of 1.2 W/cm^2^ combined with 100 μl/ml MBs) or UTMD-H (ultrasonic intensity of 1.2 W/cm^2^ combined with 200 μl/ml MBs) at a duty cycle of 10% for 60 s. Then, the cells were exposed to IR (2 Gy), and 24 h later, the level of LC3B2 was detected by Western blotting analysis and the indicated protein was quantified. **c** GL261 cells were transfected with a plasmid expressing GFP-LC3B. After 24 h, the cells were pretreated with UTMD (ultrasonic intensity of 1.2 W/cm^2^ combined with 200 μl/ml MBs at a duty cycle of 10% for 60 s). Then, the cells were exposed to IR (2 Gy) and incubated for an additional 24 h. Following fixation, the cells were immediately visualized by confocal microscopy, and the number of GFP-LC3B dots in each cell was counted. **d** GL261 cells were pretreated with UTMD (ultrasonic intensity of 1.2 W/cm^2^ combined with 200 μl/ml MBs at a duty cycle of 10% for 60 s). Then, the cells were exposed to IR (2 Gy) and incubated for another 24 h. Autophagosomes were detected by transmission electron microscopy. Arrows indicate the autophagosomes. Values are expressed as the mean ± SD (*n* = 3). *P < 0.05 vs. the vehicle-treated control group; ^#^*P* < 0.05, ^##^*P* < 0.01 vs. IR group. IR ionizing radiation, UTMD ultrasound-triggered microbubble destruction, LC3B light chain 3 beta, ACTB β-actin, GFP green fluorescent protein, MBs microbubbles, SD standard deviation, a.u. arbitrary units

As shown in Fig. 2a, b, the ratio of LC3B2 to ACTB of IR plus UTMD group (1.4 ± 0.1 or 1.4 ± 0.2 in 200 μl/ml MBs treated GL261 or U251 cells, respectively) was higher than that of IR group (0.9 ± 0.1 in GL261 cells or 1.0 ± 0.1 in U251 cells, *P* < 0.05 or *P* < 0.01). IR plus UTMD notably increased the number of GFP-LC3B dots (77 ± 8 vs. 26 ± 6, *P* < 0.01) and autophagosomes (59 ± 7 vs. 19 ± 3, *P* < 0.01) compared with IR group in GL261 cells (Fig. 2c, d). These results suggested that UTMD increased the number of autophagosomes in IR-treated glioblastoma cells.

**Fig. 3:**
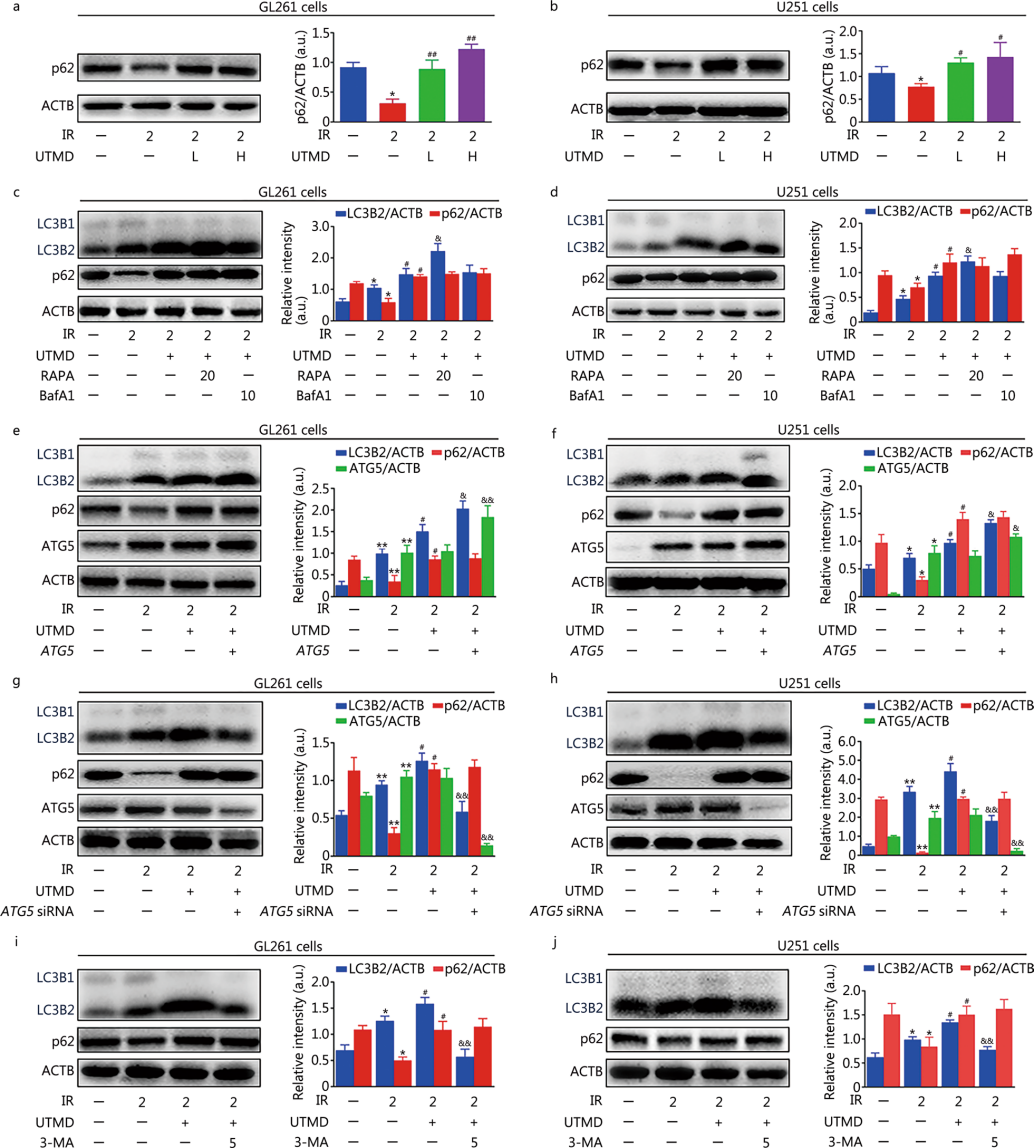
UTMD inhibited autophagic flux in IR-treated glioblastoma cells. **a**, **b** Representative immunoblot and quantification analysis of p62 after GL261 and U251 cells were treated with UTMD-L (ultrasonic intensity of 1.2 W/cm^2^ combined with 100 μl/ml MBs) or UTMD-H (ultrasonic intensity of 1.2 W/cm^2^ combined with 200 μl/ml MBs) at a duty cycle of 10% for 60 s followed by IR (2 Gy) exposure. ACTB was used as an internal standard for protein loading. **c**, **d** GL261 and U251 cells were pretreated with RAPA (20 nmol/L) or BafA1 (10 nmol/L) for 1 h and then cells were treated with UTMD (ultrasonic intensity of 1.2 W/cm^2^ combined with 200 μl/ml MBs at a duty cycle of 10% for 60 s) followed by IR (2 Gy) exposure. Total cell lysates were immunoblotted with anti-p62, anti-LC3B, and anti-ACTB antibodies and the bar graphs show the quantification of LC3B2 and p62. **e–h** GL261 and U251 cells were transfected with lentiviral vectors encoding *ATG5* or *ATG5* siRNA*.* Then, the cells were treated with UTMD (ultrasonic intensity of 1.2 W/cm^2^ combined with 200 μl/ml MBs at a duty cycle of 10% for 60 s) followed by IR (2 Gy) exposure. The expression of LC3B2, p62 and ATG5 was detected by Western blotting. The bar graphs show the quantification of LC3B2, p62 and ATG5. **i**, **j** GL261 and U251 cells were pretreated with 3-MA (5 mmol/L) for 1 h and then exposed to UTMD (ultrasonic intensity of 1.2 W/cm^2^ combined with 200 μl/ml MBs at a duty cycle of 10% for 60 s) followed by IR (2 Gy) exposure. Total cell lysates were immunoblotted with anti-p62, anti-LC3B, and anti-ACTB antibodies. The bar graphs show the quantification of LC3B2 and p62. Values are expressed as the mean ± SD (*n* = 3). *P < 0.05, **P < 0.01 vs. the vehicle-treated control group; ^#^*P* < 0.05, ^##^*P* < 0.01 vs. IR group; ^&^*P* < 0.05, ^&&^*P* < 0.05 vs. IR plus UTMD group. IR ionizing radiation, UTMD ultrasound-triggered microbubble destruction, ACTB β-actin, LC3B light chain 3 beta, p62 sequestosome 1, RAPA rapamycin, BafA1 bafilomycin A1, 3-MA 3-methyladenine, GFP green fluorescent protein, MBs microbubbles, SD standard deviation, a.u. arbitrary units

Moreover, p62 levels of IR plus UTMD group (1.2 ± 0.1 or 1.4 ± 0.3 in 200 μl/ml MBs treated GL261 or U251 cells, respectively) were higher than those of IR group (0.3 ± 0.1 in GL261 cells or 0.7 ± 0.1 in U251 cells, *P* < 0.05 or* P* < 0.01, Fig. 3a, b), indicating the suppression of autophagic degradation. Challenge with BafA1 failed to induce further accumulation of LC3B2 or p62 in IR plus UTMD treated glioblastoma cells (*P* > 0.05, Fig. 3c, d). These results indicated that UTMD inhibited cellular autophagic flux in IR-treated glioblastoma cells. As shown in Fig. 3c–f, RAPA or ATG5 overexpression markedly increased UTMD-induced LC3B2 expression (*P* < 0.05) and had no obvious effect on p62 levels (*P* > 0.05). Meanwhile, *ATG5* siRNA or 3-MA notably inhibited UTMD-induced LC3B2 accumulation (*P* < 0.01) and also had no significant effect on p62 degradation (*P* > 0.05, Fig. 3g–j). These results suggested that UTMD did not block any stage prior to autophagosome synthesis; in contrast, it inhibited the degradation of autophagic contents in IR-treated glioblastoma cells.

**Fig. 4:**
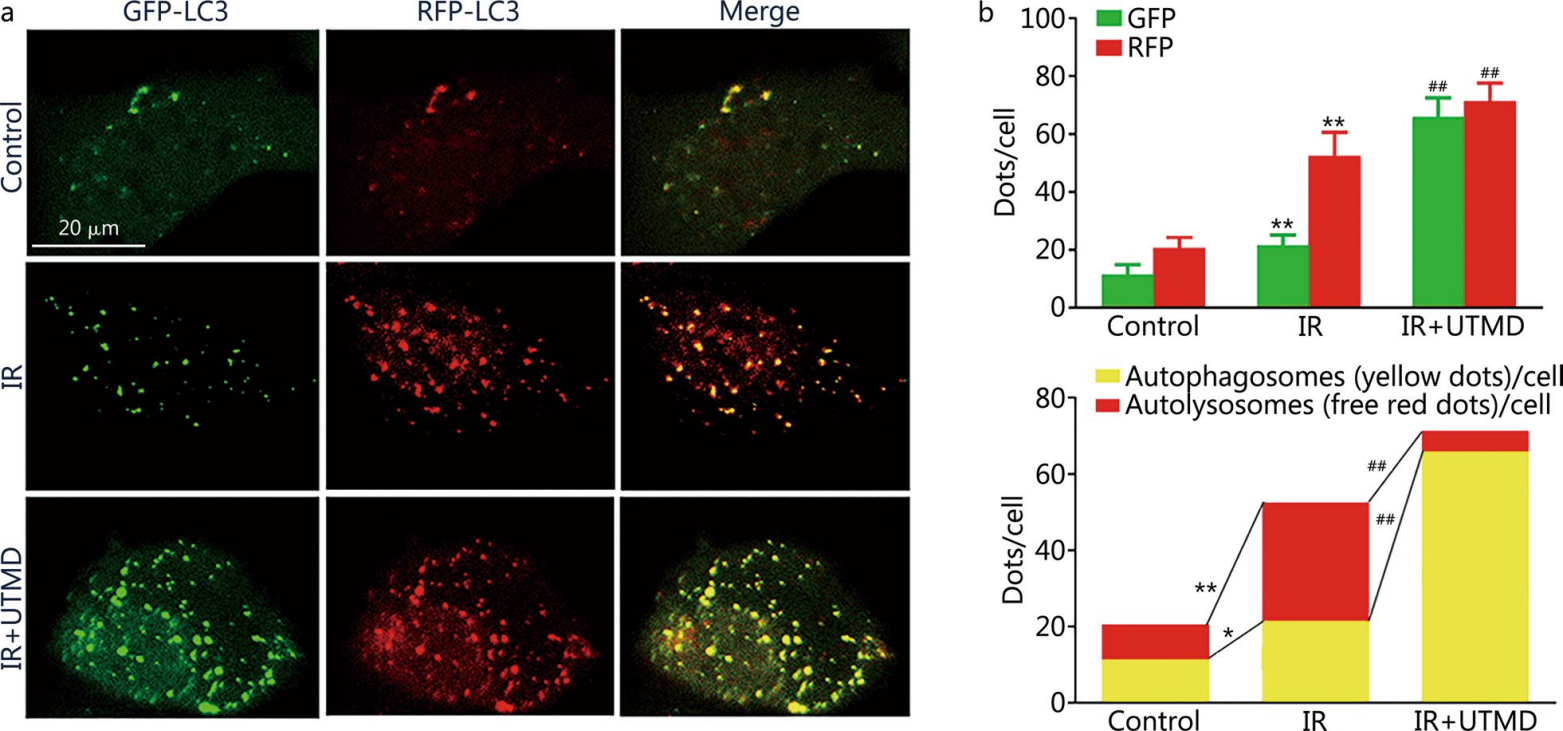
UTMD inhibited autophagosome fusion with lysosomes in IR-treated glioblastoma cells. GL261 cells were transfected with RFP-GFP-LC3B for 24 h and then exposed to UTMD (ultrasonic intensity of 1.2 W/cm^2^ combined with 200 μl/ml MBs at a duty cycle of 10% for 60 s) followed by IR (2 Gy) exposure. **a** Representative images of fluorescent LC3 puncta are shown. **b** Mean numbers of GFP and RFP dots/cell (top). Mean number of autophagosomes (yellow dots in merged images) and autolysosomes (red dots in merged images)/cell (bottom). Values are expressed as mean ± SD (*n* = 3). *P < 0.05, **P < 0.01 vs. the vehicle-treated control group; ^##^*P* < 0.01 vs. IR group. GFP green fluorescent protein, RFP red fluorescent protein, LC3B light chain 3 beta, IR ionizing radiation, UTMD ultrasound-triggered microbubble destruction, MBs microbubbles, SD standard deviation

To document whether UTMD prevented autophagosomes from reaching lysosomes, RFP-GFP-LC3 staining was used. As shown in Fig. 4a, b, the GFP (66 ± 7) dots and RFP (71 ± 6) dots of IR plus UTMD group were significantly increased compared with those of IR group [GFP (22 ± 4) dots and RFP (52 ± 8) dots, *P* < 0.01]. In the merged images, the red/yellow dots were notably decreased in IR plus UTMD group compared with those of IR group (0.08 ± 0.04 vs. 1.44 ± 0.45, *P* < 0.01), indicating marked inhibition of autolysosome formation vs. autophagosome formation and suggesting that UTMD decreased autophagosome-lysosome fusion in IR-treated glioblastoma cells.

Finally, the effect of UTMD on lysosome function was examined. UTMD had no effect on the LysoSensor green DND-189 fluorescence intensity (*P* > 0.05, Additional file 1: Fig. S3a), suggesting that the lysosomal pH was not changed in IR-treated glioblastoma cells. As shown in Additional file 1: Fig. S3b-d, UTMD did not change the fluorescence intensity of cells stained with LTG fluorescent dye or LAMP1 and LAMP2 expression (*P* > 0.05). Moreover, CTSB and CTSD activities were also not affected by UTMD in IR-treated glioblastoma cells (*P* > 0.05, Additional file 1: Fig. S3e-f). These results indicated that lysosomal function was not changed in IR plus UTMD-cotreated glioblastoma cells.

### UTMD-induced radiosensitization of glioblastoma cells by inhibiting autophagy

**Fig. 5:**
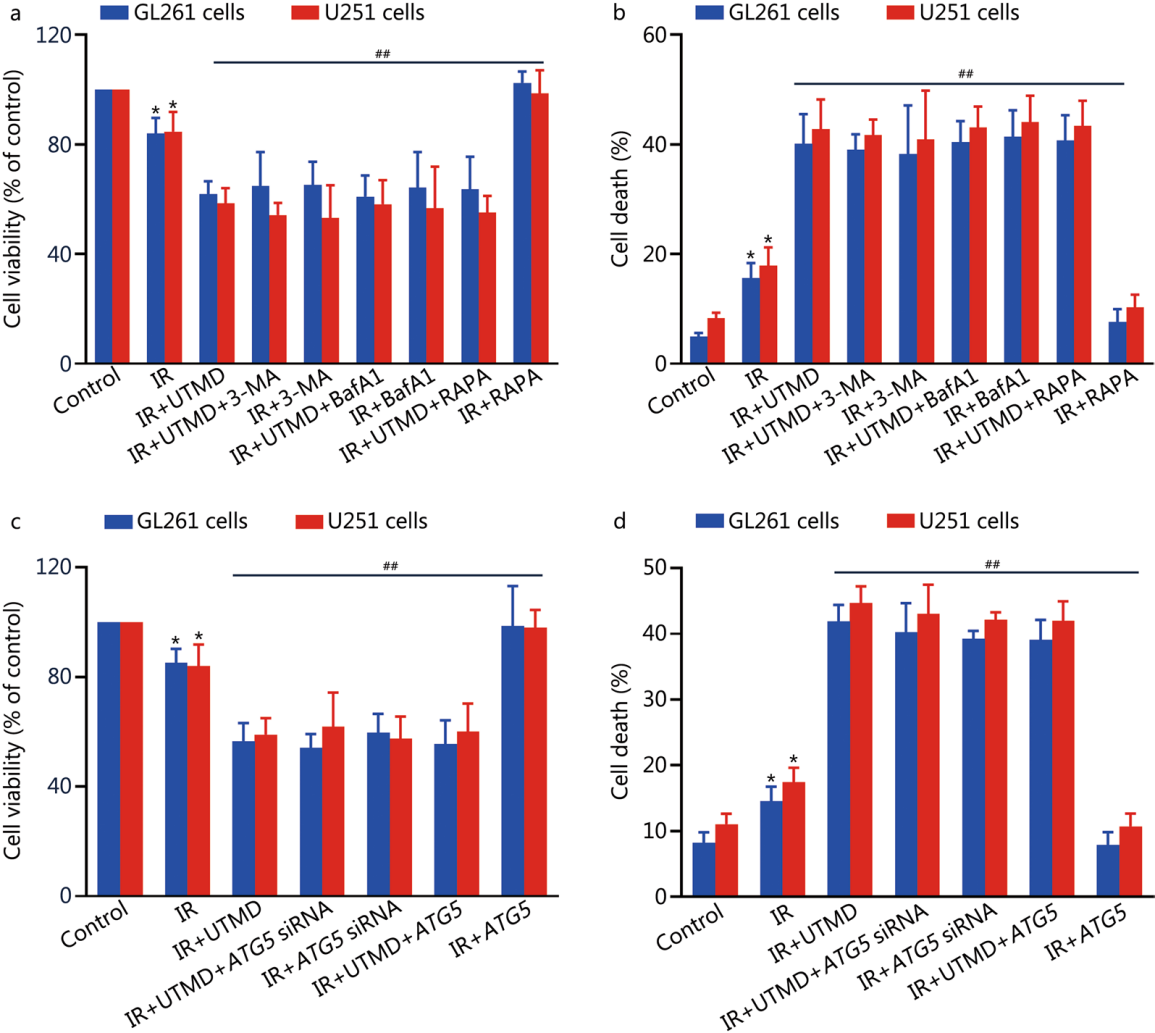
UTMD-induced radiosensitization of glioblastoma cells by inhibiting autophagy. GL261 and U251 cells were pretreated with 3-MA (5 mmol/L), BafA1 (10 nmol/L) or RAPA (20 nmol/L) for 1 h and then cells were treated with or without UTMD (ultrasonic intensity of 1.2 W/cm^2^ combined with 200 μl/ml MBs at a duty cycle of 10% for 60 s) followed by IR (2 Gy) exposure. **a** Cell viability was assessed with a CCK-8 detection kit. **b** Cell death was detected by PI staining followed by flow cytometry. GL261 and U251 cells were transfected with lentiviral vectors encoding *ATG5* or *ATG5* siRNA. Then, the cells were treated with or without UTMD (ultrasonic intensity of 1.2 W/cm^2^ combined with 200 μl/ml MBs at a duty cycle of 10% for 60 s) followed by IR (2 Gy) exposure. **c** Cell viability was assessed with a CCK-8 detection kit. **d** Cell death was detected by PI staining followed by flow cytometry. Values are expressed as mean ± SD (*n* = 3). *P < 0.05 vs. the vehicle-treated control group; ^##^*P* < 0.01 vs. IR group. IR ionizing radiation, UTMD ultrasound-triggered microbubble destruction, 3-MA 3-methyladenine, BafA1 bafilomycin A1, RAPA rapamycin, MBs microbubbles, CCK-8 cell counting kit-8, PI propidium iodide, SD standard deviation

As shown in Fig. 5a, b and Additional file 1: Fig. S2, UTMD induced an obvious increase in cell death and decrease in colony formation in IR-treated glioblastoma cells (*P* < 0.01); moreover, in the presence of 3-MA, BafA1 or RAPA, there was no significant effect on UTMD-induced cell death and inhibition of colony formation in IR-treated glioblastoma cells (*P* > 0.05). To further confirm these results, lentiviral vectors encoding the *ATG5* gene and *ATG5* siRNA were applied. As expected, we found that ATG5 overexpression or knockdown did not affect UTMD-induced cell death or reduction of colony formation in IR-treated glioblastoma cells (*P* > 0.05, Fig. 5c, d and Additional file 1: Fig. S2).

### UTMD inhibited autophagy in a PGRMC1-dependent manner in IR-treated glioblastoma cells

**Fig. 6:**
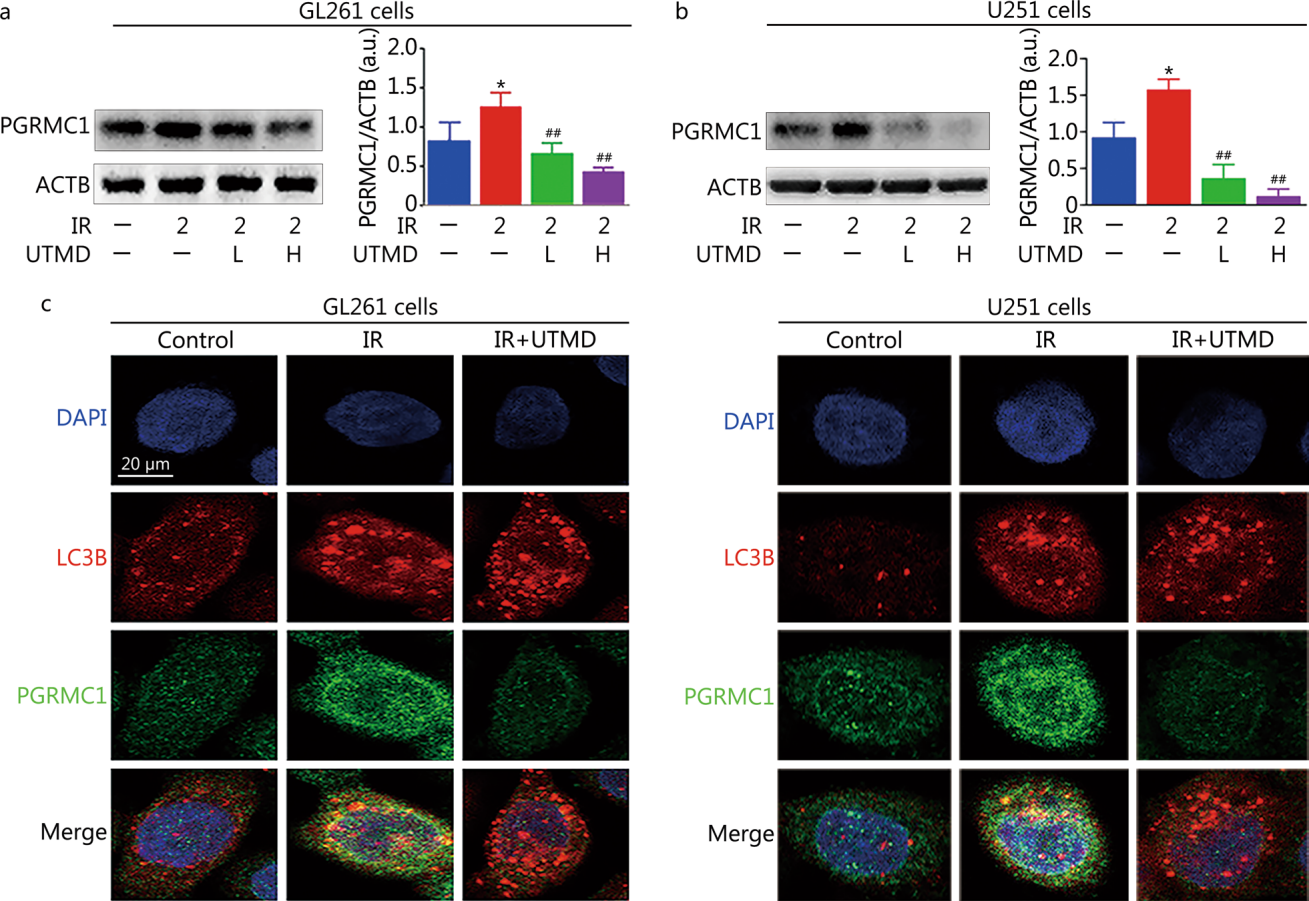
UTMD inhibited PGRMC1 expression and binding with LC3B in IR-treated glioblastoma cells. **a**, **b** GL261 and U251 cells were treated with UTMD-L (ultrasonic intensity of 1.2 W/cm^2^ combined with 100 μl/ml MBs) or UTMD-H (ultrasonic intensity of 1.2 W/cm^2^ combined with 200 μl/ml MBs), and then the cells were exposed to IR (2 Gy). PGRMC1 expression was measured by Western blotting. **c** GL261 and U251 cells were pretreated with UTMD. Then, the cells were exposed to IR (2 Gy) and incubated for an additional 24 h. Immunofluorescence staining analysis of PGRMC1 (green) and LC3B (red) expression, the nuclei were stained with DAPI (blue). Values are expressed as mean ± SD (*n* = 3). *P < 0.05 vs. the vehicle-treated control group; ^##^*P* < 0.01 vs. IR group. IR ionizing radiation, UTMD ultrasound-triggered microbubble destruction, PGRMC1 progesterone receptor membrane component 1, ACTB β-actin, LC3B light chain 3 beta, MBs microbubbles, SD standard deviation, a.u. arbitrary units

**Fig. 7:**
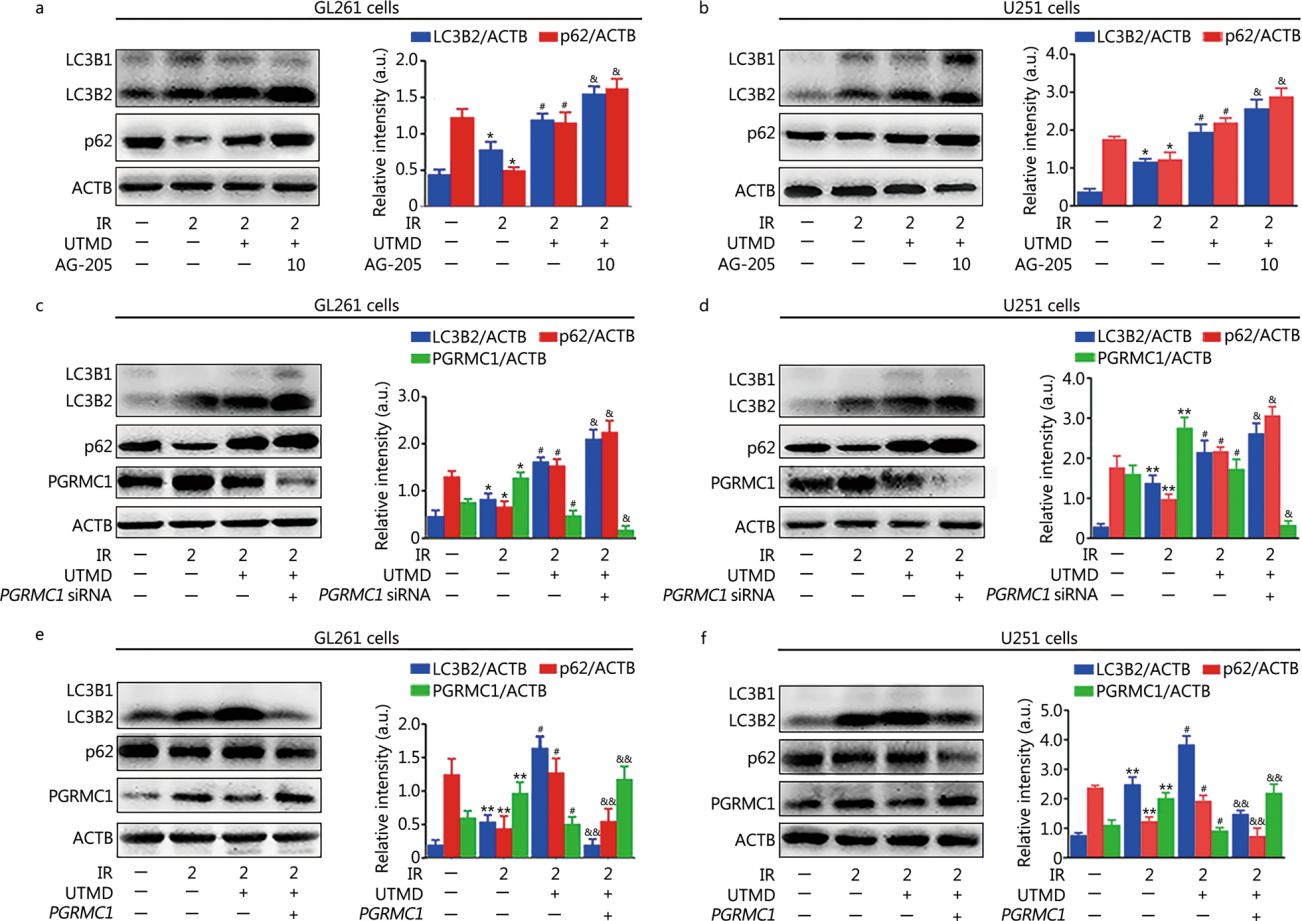
UTMD inhibited autophagy in a PGRMC1-dependent manner in IR-treated glioblastoma cells. **a**, **b** GL261 and U251 cells were pretreated with AG-205 (10 μmol/L) for 1 h and then cells were treated with UTMD followed by IR (2 Gy) exposure. After 24 h, cells were collected and lysed, then Western blotting analysis was performed. The bar graphs show the quantification of the indicated proteins. GL261 and U251 cells were transfected with lentiviral vectors encoding *PGRMC1* or *PGRMC1* siRNA. Then, the cells were treated with UTMD followed by IR (2 Gy) exposure. **c–f** The expression of LC3B2, p62, PGRMC1 and ACTB was detected by Western blotting. The bar graph shows the quantification of the indicated proteins. Values are expressed as mean ± SD (*n* = 3). *P < 0.05, **P < 0.01 vs. the vehicle-treated control group; ^#^*P* < 0.05 vs. IR group; ^&^*P* < 0.05, ^&&^*P* < 0.01 vs. IR plus UTMD group. IR ionizing radiation, UTMD ultrasound-triggered microbubble destruction, LC3B light chain 3 beta, p62 sequestosome 1, ACTB β-actin, PGRMC1 progesterone receptor membrane component 1, SD standard deviation, a.u. arbitrary units

**Fig. 8:**
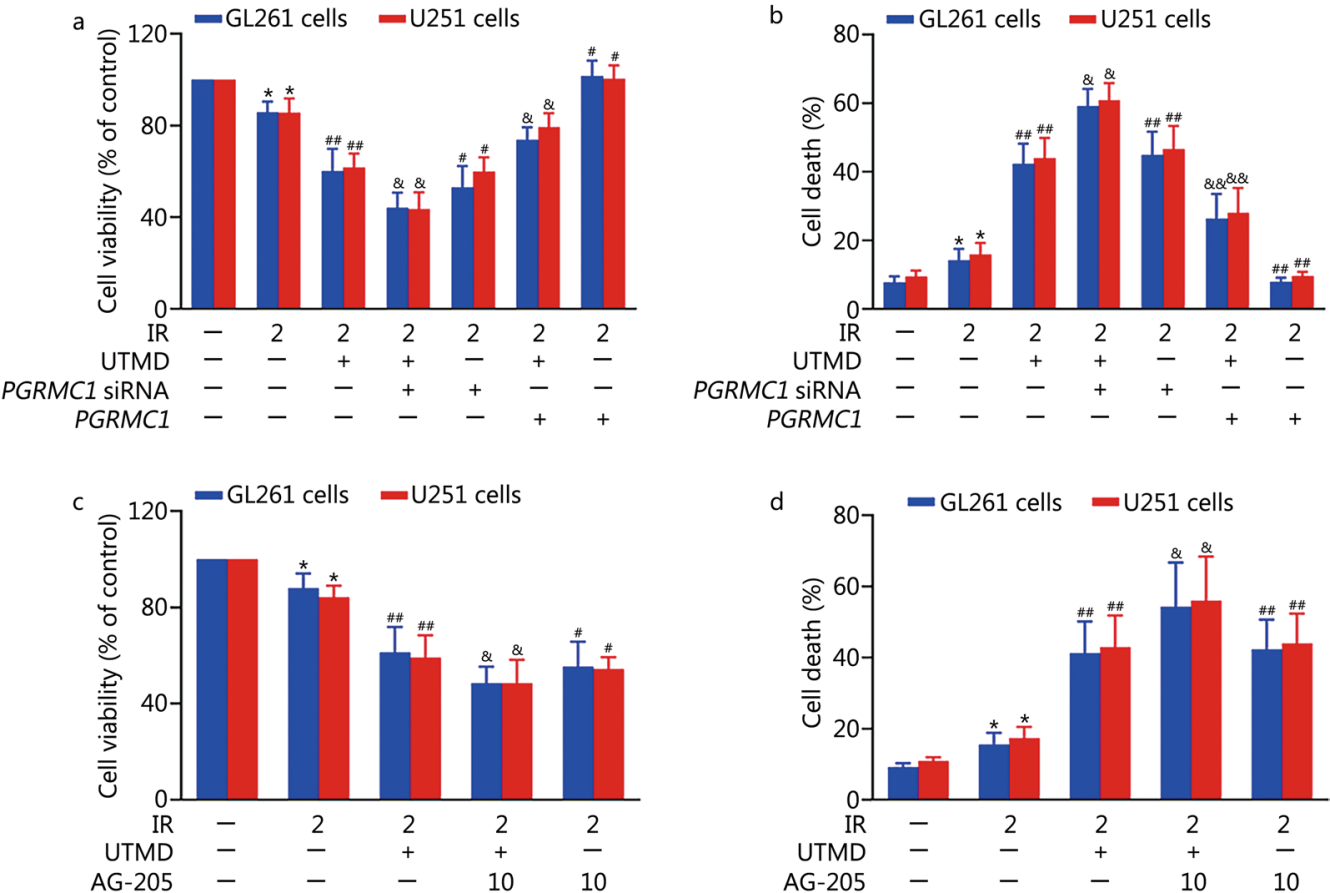
UTMD induced radiosensitization of glioblastoma cells in a PGRMC1-dependent manner. GL261 and U251 cells were transfected with lentiviral vectors encoding *PGRMC1* or *PGRMC1* siRNA. Then, the cells were treated with UTMD followed by IR (2 Gy) exposure. **a** Cell viability was assessed with a CCK-8 detection kit. **b** Cell death was detected by PI staining followed by flow cytometry. GL261 and U251 cells were pretreated with AG-205 (10 μmol/L) for 1 h and then cells were treated with UTMD followed by IR (2 Gy) exposure.** c** Cell viability was assessed with a CCK-8 detection kit. **d** Cell death was detected by PI staining followed by flow cytometry. Values are expressed as mean ± SD (*n* = 3). *P < 0.05 vs. the vehicle-treated control group; ^#^*P* < 0.05, ^##^*P* < 0.01 vs. IR group; ^&^*P* < 0.05, ^&&^*P* < 0.01 vs. IR plus UTMD group. IR ionizing radiation, UTMD ultrasound-triggered microbubble destruction, PGRMC1 progesterone receptor membrane component 1, CCK-8 cell counting kit-8, PI propidium iodide, SD standard deviation

As indicated in Fig. 6a, b, IR plus UTMD inhibited PGRMC1 expression (0.4 ± 0.1 or 0.1 ± 0.1 in 200 μl/ml MBs treated GL261 or U251 cells, respectively) compared with IR group (1.3 ± 0.2 in GL261 cells or 1.6 ± 0.1 in U251 cells, *P* < 0.01). Meanwhile, the colocalization of PGRMC1 and LC3B2 was also markedly decreased by UTMD in IR-exposed glioblastoma cells (Fig. 6c). Moreover, AG-205 or *PGRMC1* siRNA pretreatment notably enhanced UTMD-induced LC3B2 and p62 accumulation in IR-exposed glioblastoma cells (*P* < 0.05, Fig. 7a–d). In contrast, PGRMC1 overexpression abolished UTMD-induced blockade of autophagic degradation in IR-treated glioblastoma cells (*P* < 0.01, Fig. 7e, f). Additionally, AG-205 or *PGRMC1* siRNA treatment notably improved UTMD-induced radiosensitization of glioblastoma cells, and PGRMC1 overexpression markedly inhibited UTMD-induced radiosensitization of glioblastoma cells (*P* < 0.05, Fig. 8a–d and Additional file 1: Fig. S2).

### UTMD enhanced the radiosensitivity of glioblastoma via inhibiting PGRMC1-mediated autophagy in mice

**Fig. 9:**
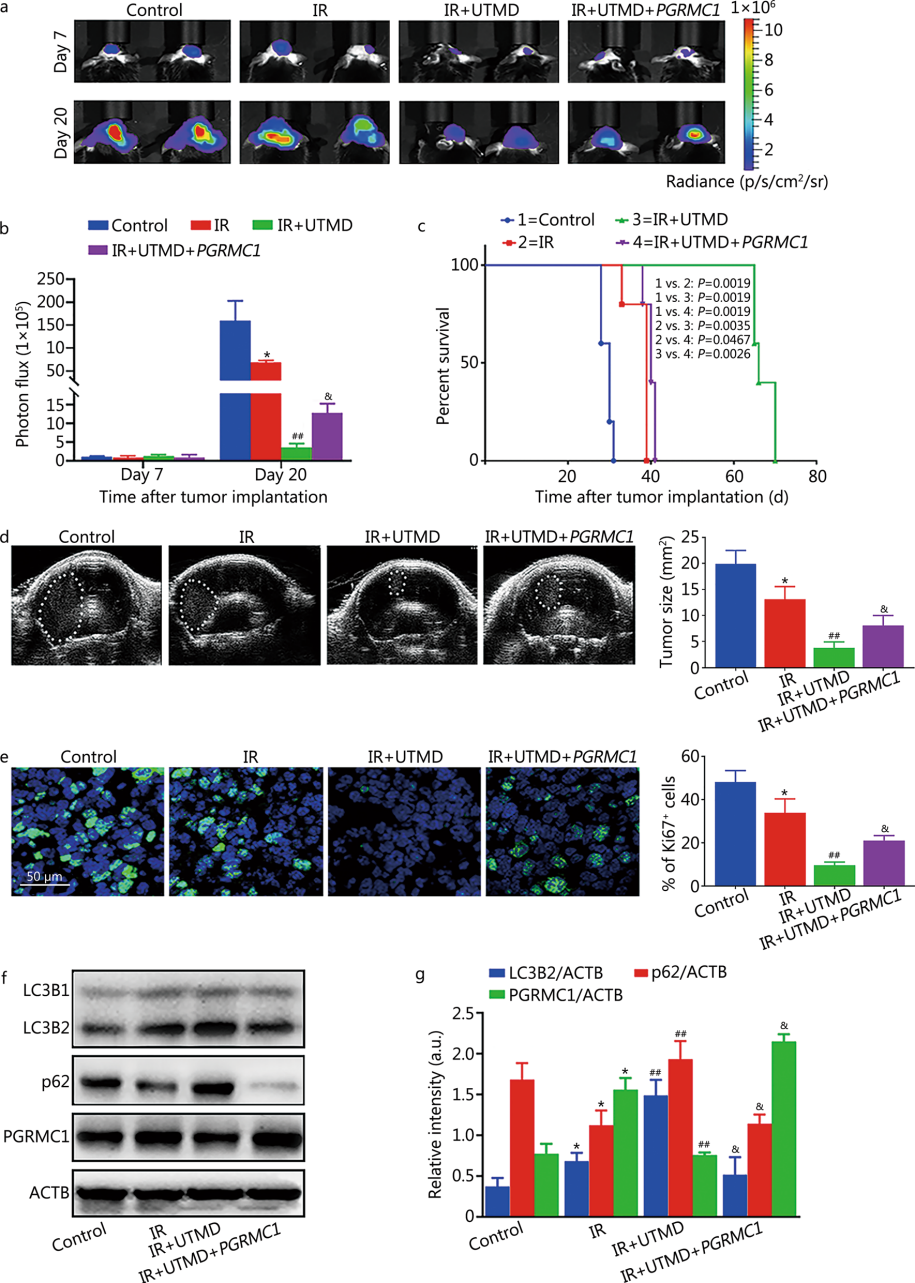
UTMD enhanced the radiosensitivity of glioblastoma via inhibiting PGRMC1-mediated autophagy in mice. Six-week-old female C57BL/6J mice were inoculated in the right caudate putamen with GL261 cells (1 × 10^5^ cells per mouse) with or without shPGRMC1-expressing vector to establish the orthotopic glioblastoma mouse model. Seven days after transplantation, the tumor size was evaluated by in vivo bioluminescent imaging. Then, the animals were randomly allocated to treatment groups to receive control treatment or IR (every day, 2 Gy/fraction, 5 fractions) combined with or without UTMD (every other day) treatment over a period of 5 d. On day 20 after tumor transplantation, US and bioluminescent imaging were used to determine the tumor size. Meanwhile, mice were euthanized, and brain tumors were collected. In vivo bioluminescent images **(a)** and quantification of orthotopic glioblastoma tumors **(b)** in the brains of mice. **c** Kaplan–Meier survival analysis of orthotopic glioblastoma-bearing mice. **d** Orthotopic glioblastoma tumor size was determined by US. White circles indicate tumors.** e** Immunofluorescent staining and the quantification of Ki67 (green) in orthotopic glioblastoma tumors in the brains of mice. **f** Tumor tissues were harvested on day 20 after tumor implantation and lysed, and Western blotting analysis was performed. The expression of LC3B2, p62, and PGRMC1 were measured. **g** The bar graphs showed the quantification of LC3B2, p62, and PGRMC1. Values are expressed as mean ± SD (*n* = 6). *P < 0.05 vs. the vehicle-treated control group; ^##^*P* < 0.01 vs. IR group; ^&^*P* < 0.05 vs. IR plus UTMD group. IR ionizing radiation, UTMD ultrasound-triggered microbubble destruction, PGRMC1 progesterone receptor membrane component 1, LC3B light chain 3 beta, p62 sequestosome 1, ACTB β-actin, SD standard deviation, p/s/cm^2^/sr photon per second per square centimetre per steradian, a.u. arbitrary units

As shown in Additional file 1: Fig. S4, IR plus UTMD (3% V/V MBs with 0.8 MI value) group had the smallest tumor size and the longest survival extension among all the experimental groups. Therefore, 3% V/V MBs with 0.8 MI value was set as the optimal parameter for treating mice in subsequent experiments to clarify the underlying mechanism of UTMD-induced enhancement of IR response in glioblastoma in vivo. As compared with IR group, IR plus UTMD significantly inhibited tumor growth [(13.1 ± 2.4) mm^2^ vs. (3.8 ± 1.1) mm^2^, *P* < 0.01] and extended survival time [(37.8 ± 2.7) d vs. (67.2 ± 2.6) d, *P* = 0.0035] of glioblastoma-bearing mice (Fig. 9a–d). Immunofluorescent staining of Ki67 showed that the Ki67 levels of the IR plus UTMD group were much lower than those of single IR-exposed glioblastoma-bearing mice [(9.6 ± 1.5)% vs. (34.0 ± 6.4)%, *P* < 0.01], indicating a significant decrease of proliferating cells in glioblastoma (Fig. 9e). These results suggested that UTMD improved the therapeutic efficacy of IR in vivo. Furthermore, we also found that UTMD inhibited autophagic flux and PGRMC1 expression in IR-treated glioblastoma-bearing mice (Fig. 9f, g). As compared with IR plus UTMD group, PGRMC1 overexpression markedly reversed UTMD-induced inhibition of autophagic flux and PGRMC1 expression, thereby abolishing the beneficial effect of UTMD on tumor size [(3.8 ± 1.1) mm^2^ vs. (8.0 ± 1.9) mm^2^, *P* < 0.05] or survival time [(67.2 ± 2.6) d vs. (40.0 ± 1.2) d, *P* = 0.0026] in glioblastoma-bearing mice, respectively (Fig. 9a–g). Collectively, our data indicated that PGRMC1-mediated autophagy played a critical role in UTMD-induced radiosensitization of glioblastoma in vivo.

## Discussion

Radiotherapy remains a major modality for glioblastoma treatment; however, the average overall survival of glioblastoma patients is poor due to the high radioresistance of glioblastoma cells [2]. Therefore, effective radiosensitizers are desperately needed. In line with previous observations [2], we found that glioblastoma cells were resistant to IR exposure. Meanwhile, 60 Gy whole brain radiation therapy in 30 fractions (2 Gy/fraction) was the most widely used dose for glioblastoma patients, and many studies focused on developing effective radiosensitizers for glioblastoma cells were used a single dose of 2 Gy for IR exposure in vitro [33, 34]. Accordingly, IR at a single dose of 2 Gy was used in our experiments in vitro and 10 Gy whole brain radiation therapy in 5 fractions (2 Gy/fraction) was chosen in vivo. UTMD is a noninvasive approach that can disrupt cell membrane integrity at a given site and induce increased capillary permeability through bursting of MBs and has important applicational value in both military and civilian medicine. During recent decades, UTMD has been considered as a promising strategy for sensitizing various malignant tumors, such as liver, bladder, prostate and breast tumors, to IR; this strategy has low invasivity, low immunogenicity, and repeatable applicability, indicating that UTMD would be a good candidate for radiosensitizer [4, 6, 35–37]. Recently, Burke et al. [8] demonstrated that UTMD is potential to increase therapeutic efficiency by enhancing the drug payload in glioma. Zhao et al. [10] also found that UTMD combined with cilengitide (CTG) nanoparticles significantly increases the tumor CGT content over threefold and prolongs tumor retention times in glioma, indicating that this method overcomes the limitations of standard CGT treatment and offers hope for bringing CGT therapy back to the clinical setting for glioma. However, relevant studies about the effect of UTMD on the radiotherapy response of glioma are currently rare. In 2021, Peng et al. [30] first confirmed that UTMD improves the destructive effect of IR on glioblastoma in a subcutaneous tumor model in nude mice, which demands to be further validated in an animal model in situ that can recapitulate various aspects of disease more accurately. Accordingly, in the present study, we established an orthotopic mouse glioblastoma model and found, for the first time, that UTMD markedly enhanced the radiosensitivity and prolonged the survival of orthotopic glioblastoma-bearing mice. Our findings are important complements to previous studies regarding the effect of UTMD on radiotherapy for tumors, especially glioma, suggesting that UTMD would be an effective adjuvant for radiotherapy in glioblastoma.

Autophagy is a cellular housekeeping process to maintain cellular homeostasis by removing damaged proteins and organelles through an alternative mechanism (i.e., non-proteasomal) degradation, which plays important role in tumorigenesis and tumor treatment [38]. Although the effect of autophagy on tumorigenesis and cancer therapy is complex, many lines of evidence have shown that radiotherapy can induce cytoprotective autophagy, which promotes therapeutic resistance. Pharmacological inhibitors or siRNAs that inhibit autophagy can sensitize resistant cancer cells to radiotherapy and specifically target autophagy-addicted tumors [39]. IR induces autophagy in glioma cells, subsequently leading to radioresistance [40], and chloroquine, an autophagy inhibitor, potentiates the radiosensitivity of glioma-initiating cells by inhibiting autophagy and activating apoptosis [41]. Meanwhile, inhibition of autophagy mediated by microRNA-17-5p [42], microRNA-93 [16], or CTSD [43] also enhances the response of glioblastoma cells to IR. These results clearly support that downregulating of IR-caused protective autophagy would bring therapeutic benefit to glioma. Moreover, US has been found to increase the clearance of neuronal tau by upregulating autophagy thereby improving behavioral functions in tau transgenic mice [44]. Recently, several studies have shown that US can induce autophagy, thereby leading to cell death in nasopharyngeal carcinoma [18] and castration-resistant prostate cancer cells [20], suggesting the potential of US-modulated autophagy as a novel therapeutic strategy for tumors. In contrast, Wang et al. [17] confirmed that US promotes MSCs chondrogenesis by inhibiting autophagy. Another study found that US induces protective autophagy in paclitaxel-resistant PC-3 cells and inhibition of US-mediated autophagy promotes the chemosensitization effect of US in prostate cancer [19]. These findings indicate a complex role of autophagy in the beneficial biological effects of US. Herein, we, for the first time, demonstrated that UTMD inhibited IR-induced autophagy by disrupting autophagosome-lysosome fusion without impairing lysosomal function or autophagosome synthesis, thereby enhancing the radiosensitivity of glioblastoma. Moreover, the inhibition or activation of autophagosome formation as well as the disruption of autophagic degradation could not protect glioblastoma cells from UTMD-induced inhibition of cell survival. The possible reason was that UTMD notably interrupted autophagic flux at the degradation stage, thereby leading to extensive cell death. Overall, we concluded that UTMD increased the radiosensitivity of glioblastoma cells by strongly blocking autophagic flux at the degradation stage, which was not “autophagic cell death”. This work provides a new insight to interpret the potential mechanism of the radiosensitization effects of UTMD, in which autophagy may play an important role. In addition, previous studies have identified that UTMD enhances the efficiency of radiotherapy via its local cavitation-based antivascular effects in liver, breast, bladder, prostate, and fibrosarcoma tumor models [4, 6, 35–37]. Whether UTMD could improve the response of glioblastoma to IR through tumor vasculature disruption remains to be explored.

Furthermore, our results supported an important role for PGRMC1 in UTMD-mediated inhibition of autophagy in IR-treated glioblastoma cells. PGRMC1 belongs to the membrane-associated progesterone receptor family is a multifunctional protein anchored to the cell membrane through the N-terminal transmembrane helix [45, 46]. PGRMC1 is highly expressed in various tumors including breast [21], head and neck cancers [23] and even in glioma [22]. It is an important biomarker of the proliferative status of cancers and a potential therapeutic target for inhibiting tumorigenesis [25, 47]. Recently, Mir et al. [26] reported that PGRMC1 plays a critical role in autophagy by directly binding with LC3B2, and PGRMC1 inhibition disrupts autophagic flux and arrests the degradation of p62 causing aberrant mitochondria accumulation. These findings suggest a new mechanism for PGRMC1-based therapeutics in tumors. Researchers have shown that PGRMC1 contributes to doxorubicin-induced chemoresistance in uterine sarcoma [4] and *PGRMC1* knockdown increases the sensitivity of colon and liver cancers to erlotinib treatment [25]. More recently, Zhu et al. [27] pointed out that hyperoside sensitizes ovarian cancer cells to cisplatin by activating PGRMC1-mediated autophagy. In our study, we found that UTMD notably decreased the expression of PGRMC1 and the colocalization of PGRMC1 and LC3B2. Meanwhile, AG-205 or *PGRMC1* siRNA pretreatment enhanced UTMD-caused disruption of autophagy, thereby promoting UTMD-caused radiosensitization of glioblastoma cells. In contrast, PGRMC1 overexpression abolished UTMD-induced blockage of autophagic degradation and tumor radiation response enhancement in glioblastoma. Previously, Ibsen et al. [48] reported that UTMD activates chemosensory neurons and elicits withdrawal behavior by activating the membrane transient receptor potential 4 mechano-sensitive channels in Caenorhabditis elegans. Herein, we found that PGRMC1, a membrane receptor, was also involved in UTMD-induced inhibition of autophagy and radiosensitivity of glioblastoma in vitro and in vivo. However, it should be noted that all our experiments were taken in mice and cells, the effect of UTMD on radiosensitivity of glioblastoma patients had not been elucidated as well as the precise mechanisms by which UTMD inhibited PGRMC1 expression and activity. These were the limitations of our present study, which need further research, especially large-scale, long-period, randomized controlled trials.

## Conclusion

**Fig. 10:**
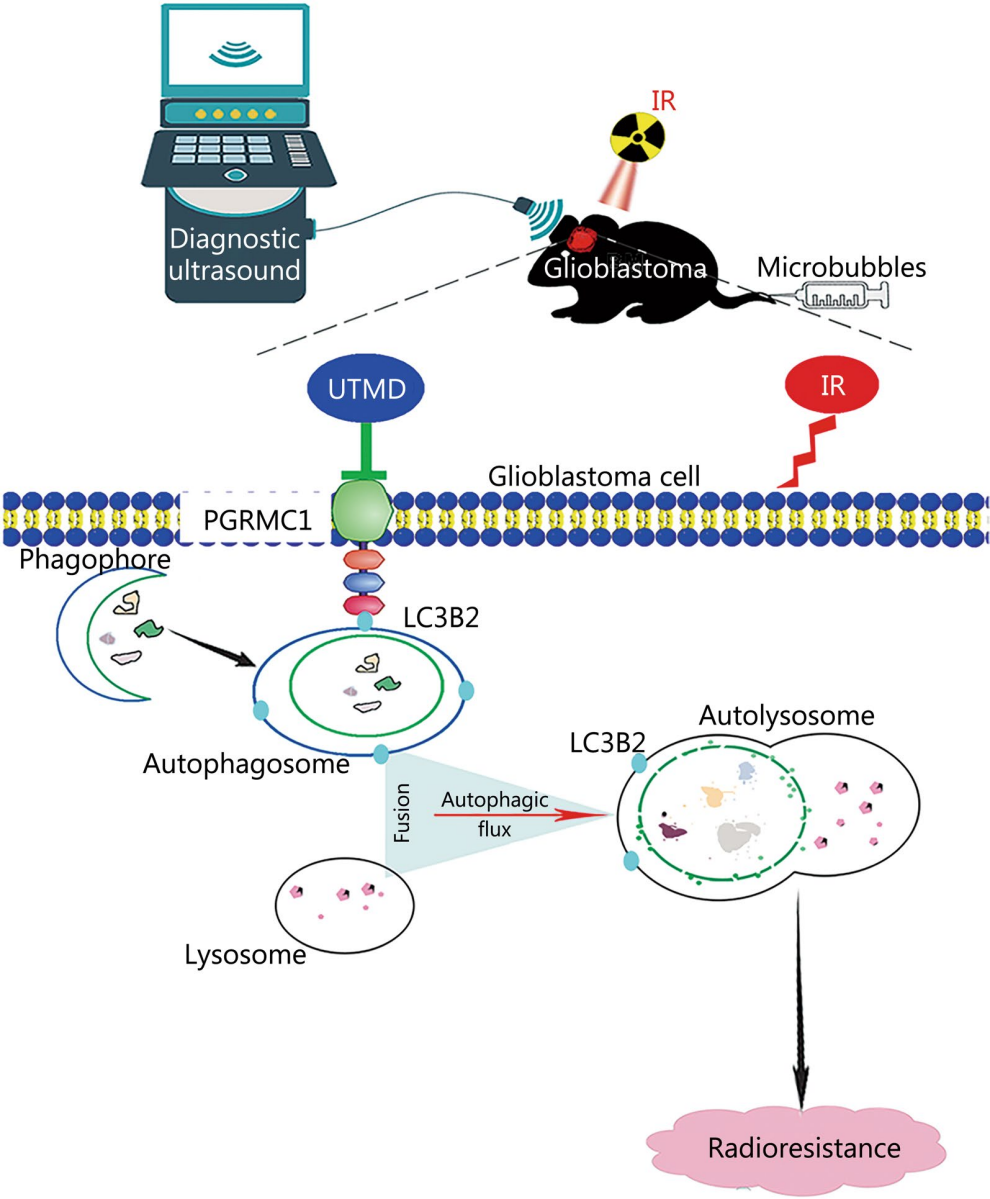
UTMD enhanced the radiosensitivity of glioblastoma by inhibiting PGRMC1-mediated autophagy. UTMD improved the response of glioblastoma cells to IR via the proposed signaling pathways: UTMD downregulates PGRMC1 expression, thereby inhibiting the combination of PGRMC1 with LC3B2, which is followed by a decrease of autophagosome-lysosome fusion, subsequently disrupting autophagic degradation and ultimately enhancing the radiosensitivity of glioblastoma. IR ionizing radiation, UTMD ultrasound-triggered microbubble destruction, PGRMC1 progesterone receptor membrane component 1, LC3B light chain 3 beta

In general, we have uncovered a novel mechanism whereby UTMD enhanced the radiosensitivity of glioblastoma through the inactivation of PGRMC1-mediated autophagy, at least partially (Fig. 10). Our collective findings provide a new insight into the mechanism of UTMD-induced promotional effects on radiotherapy and are an important complement to previous studies. These results indicate that UTMD would be an effective adjuvant for radiotherapy in glioblastoma and provide new biological evidence for clinical applications of UTMD in tumor therapy.

## Supplementary Information

**Additional file 1.** **Fig. S1.** **Schematic diagram of glioblastoma treatments**. **a** Glioblastoma cells were treated with IR and UTMD in vitro. **b** Glioblastoma-bearing mice were treated with IR and UTMD in vivo. IR ionizing radiation, UTMD ultrasound-triggered microbubble destruction, 3-MA 3-methyladenine, BafA1 bafilomycin A1, PGRMC1 progesterone receptor membrane component 1, CTSB cathepsin B, CTSD cathepsin D, RAPA rapamycin, MBs microbubbles, GFP green fluorescent protein, RFP red fluorescent protein, CCK-8 cell counting kit-8, PI propidium iodide, LTG LysoTracker green fluorescent dye, US ultrasound. **Fig. S2.** Colony formation of glioblastoma cells was measured by clonogenic assay. GL261 and U251 cells were treated with 3-MA (5 mmol/L), BafA1 (10 nmol/L), AG-205 (10 µmol/L), or RAPA (20 nmol/L) for 1 h followed by IR (2 Gy) or IR plus UTMD treatment for another 24 h. Moreover, GL261 and U251 cells were transfected with lentiviral vectors encoding *AGT5* or *PGRMC1*. Then, cells were treated with UTMD followed by IR (2 Gy) exposure for another 24 h. The conlony formation of glioblastoma cells was measured by clonogenic assay. Values are expressed as mean ± SD (*n* = 3). *P < 0.05 vs. the vehicle-treated control group; ^##^*P* < 0.01 vs. IR group; ^&&^*P* < 0.01 vs. IR plus UTMD group. IR ionizing radiation, UTMD ultrasound-triggered microbubble destruction, 3-MA 3-methyladenine, BafA1 bafilomycin A1, RAPA rapamycin, PGRMC1 progesterone receptor membrane component 1, SD standard deviation. **Fig. S3.** UTMD had no effect on lysosomal function in IR-treated glioblastoma cells. GL261 and U251 cells were treated with UTMD-L (ultrasonic intensity of 1.2 W/cm^2^ combined with 100 μl/ml MBs) or UTMD-H (ultrasonic intensity of 1.2 W/cm^2^ combined with 200 μl/ml MBs) at a duty cycle of 10% for 60 s, and then the cells were exposed to IR (2 Gy). **a** LysoSensor DND-189 fluorescence intensity was quantified using an Infinite™ M200 Microplate Reader (Tecan Group Ltd.). **b** LTG fluorescence intensity was measured by a ZEISS LSM 900 confocal laser scanning microscope. **c-d** The expression of LAMP1, LAMP2 and ACTB was detected by Western blotting. The bar graph shows the quantification of the indicated proteins. **e–f** CTSB and CTSD activity was determined by a CTSB or CTSD activity kit, respectively. Values are expressed as mean ± SD (*n* = 3). *P < 0.05, **P < 0.01 vs. the vehicle-treated control group. IR ionizing radiation, UTMD ultrasound-triggered microbubble destruction, LTG LysoTracker Green fluorescent dye, LAMP lysosomal-associated membrane protein, CTSB cathepsin B, CTSD cathepsin D, ACTB β-actin, MBs microbubbles, SD standard deviation, a.u. arbitrary units. **Fig. S4.** Optimizing the parameters of UTMD to enhance the IR response of glioblastoma in vivo. Six-week-old female C57BL/6J mice were inoculated in the right caudate putamen with GL261 cells (1 × 10^5^ cells per mouse) to establish the orthotopic glioblastoma mouse model. Seven days after transplantation, the tumor size was evaluated by in vivo bioluminescent imaging. Then, the animals were randomly allocated to treatment groups to receive control treatment or IR (everyday, 2 Gy/fraction, 5 fractions) combined with or without UTMD (every other day) treatment over a period of 5 d. On day 20 after tumor transplantation, bioluminescent imaging was used to determine the tumor size. **a** In vivo bioluminescent images (*n* = 6). **b** Kaplan–Meier survival analysis of orthotopic glioblastoma-bearing mice (*n* = 6). IR ionizing radiation, UTMD ultrasound-triggered microbubble destruction, MBs microbubbles, p/s/cm^2^/sr photon per second per square centimetre per steradian.

## Data Availability

All data and materials are available to the researchers once published.

## Abbreviations

BafA1: Bafilomycin A1
CCK-8: Cell counting kit-8
CTG: Cilengitide
CTSB: Cathepsin B
CTSD: Cathepsin D
DMEM: Dulbecco’s Modified Eagle’s Medium
GFP: Green fluorescent protein
IR: Ionizing radiation
LAMP: Lysosomal-associated membrane protein
LC3B2: Light chain 3 beta 2
LTG: LysoTracker green fluorescent dye
3-MA: 3-Methyladenine
MBs: Microbubbles
MSCs: Mesenchymal stem cells
mTOR: Mammalian target of rapamycin
PBS: Phosphate-buffered saline
PGRMC1: Progesterone receptor membrane component 1
PI: Propidium iodide
RAPA: Rapamycin
RFP: Red fluorescent protein
SQSTM1/p62: Sequestosome 1
SD: Standard deviation
US: Ultrasound
UTMD: Ultrasound-triggered microbubble destruction
