# Supplementary material for: Ultrasound-triggered microbubble destruction enhances the radiosensitivity of glioblastoma by inhibiting PGRMC1-mediated autophagy in vitro and in vivo
Source: Mil Med Res. 2022 Feb 14;9:9. doi: 10.1186/s40779-022-00369-0 (PMC8842919; doi:10.1186/s40779-022-00369-0)
Supplement: Supplementary file 1 — Additional file 1. Fig. S1. Schematic diagram of glioblastoma treatments. a Glioblastoma cells were treated with IR and UTMD in vitro. b Glioblastoma-bearing mice were treated with IR and UTMD in vivo. IR ionizing radiation, UTMD ultrasound-triggered microbubble destruction, 3-MA 3-methyladenine, BafA1 bafilomycin A1, PGRMC1 progesterone receptor membrane component 1, CTSB cathepsin B, CTSD cathepsin D, RAPA rapamycin, MBs microbubbles, GFP green fluorescent protein, RFP red fluorescent protein, CCK-8 cell counting kit-8, PI propidium iodide, LTG LysoTracker green fluorescent dye, US ultrasound. Fig. S2. Colony formation of glioblastoma cells was measured by clonogenic assay. GL261 and U251 cells were treated with 3-MA (5 mmol/L), BafA1 (10 nmol/L), AG-205 (10 µmol/L), or RAPA (20 nmol/L) for 1 h followed by IR (2 Gy) or IR plus UTMD treatment for another 24 h. Moreover, GL261 and U251 cells were transfected with lentiviral vectors encoding AGT5 or PGRMC1. Then, cells were treated with UTMD followed by IR (2 Gy) exposure for another 24 h. The conlony formation of glioblastoma cells was measured by clonogenic assay. Values are expressed as mean ± SD (n = 3). *P < 0.05 vs. the vehicle-treated control group; ##P < 0.01 vs. IR group; &&P < 0.01 vs. IR plus UTMD group. IR ionizing radiation, UTMD ultrasound-triggered microbubble destruction, 3-MA 3-methyladenine, BafA1 bafilomycin A1, RAPA rapamycin, PGRMC1 progesterone receptor membrane component 1, SD standard deviation. Fig. S3. UTMD had no effect on lysosomal function in IR-treated glioblastoma cells. GL261 and U251 cells were treated with UTMD-L (ultrasonic intensity of 1.2 W/cm2 combined with 100 μl/ml MBs) or UTMD-H (ultrasonic intensity of 1.2 W/cm2 combined with 200 μl/ml MBs) at a duty cycle of 10% for 60 s, and then the cells were exposed to IR (2 Gy). a LysoSensor DND-189 fluorescence intensity was quantified using an Infinite™ M200 Microplate Reader (Tecan Group Ltd.). b LTG fluorescence intensity was measur [file 40779_2022_369_MOESM1_ESM.pdf]

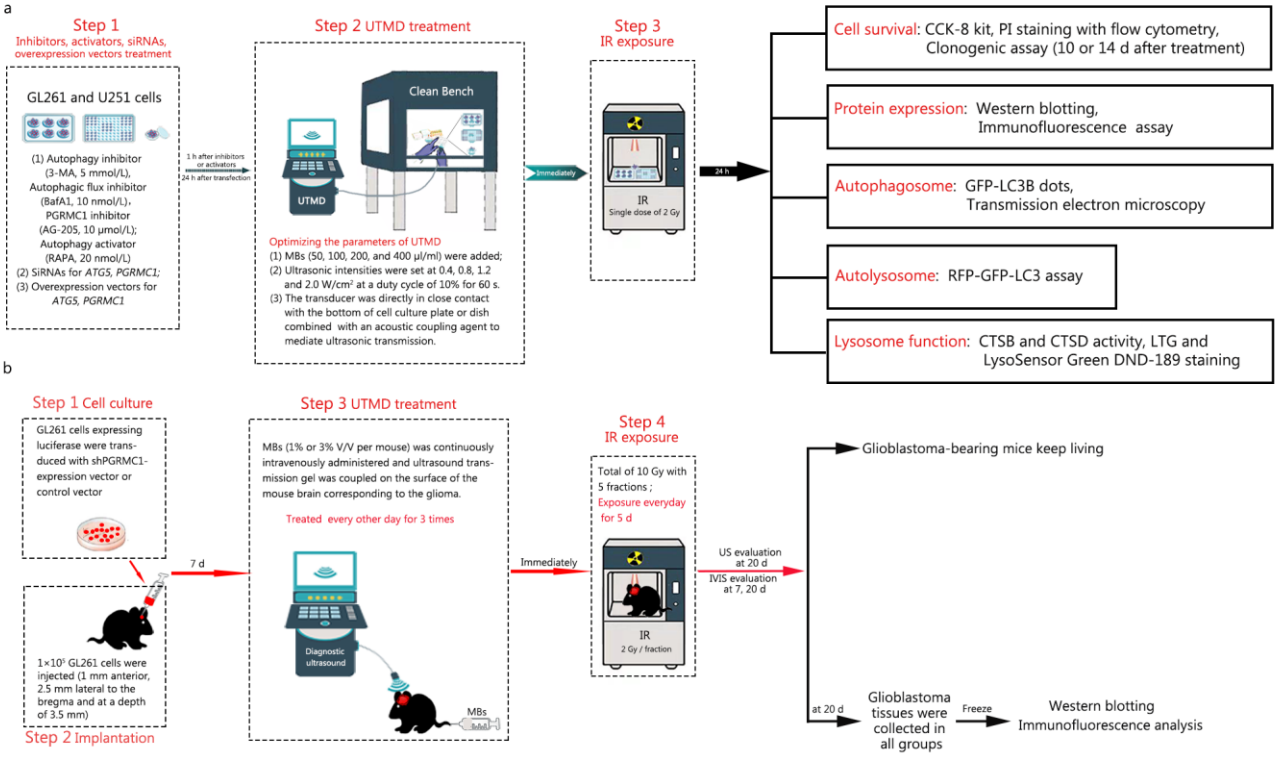

Fig. S1 Schematic diagram of glioblastoma treatments. a Glioblastoma cells were treated with IR and UTMD in vitro. **b** Glioblastoma-bearing mice were treated with IR and UTMD in vivo. IR ionizing radiation, UTMD ultrasound-triggered microbubble destruction, 3-MA 3-methyladenine, BafA1 bafilomycin A1, PGRMC1 progesterone receptor membrane component 1, CTSB cathepsin B, CTSD cathepsin D, RAPA rapamycin, MBs microbubbles, GFP green fluorescent protein, RFP red fluorescent protein, CCK-8 cell counting kit-8, PI propidium iodide, LTG LysoTracker green fluorescent dye, US ultrasound

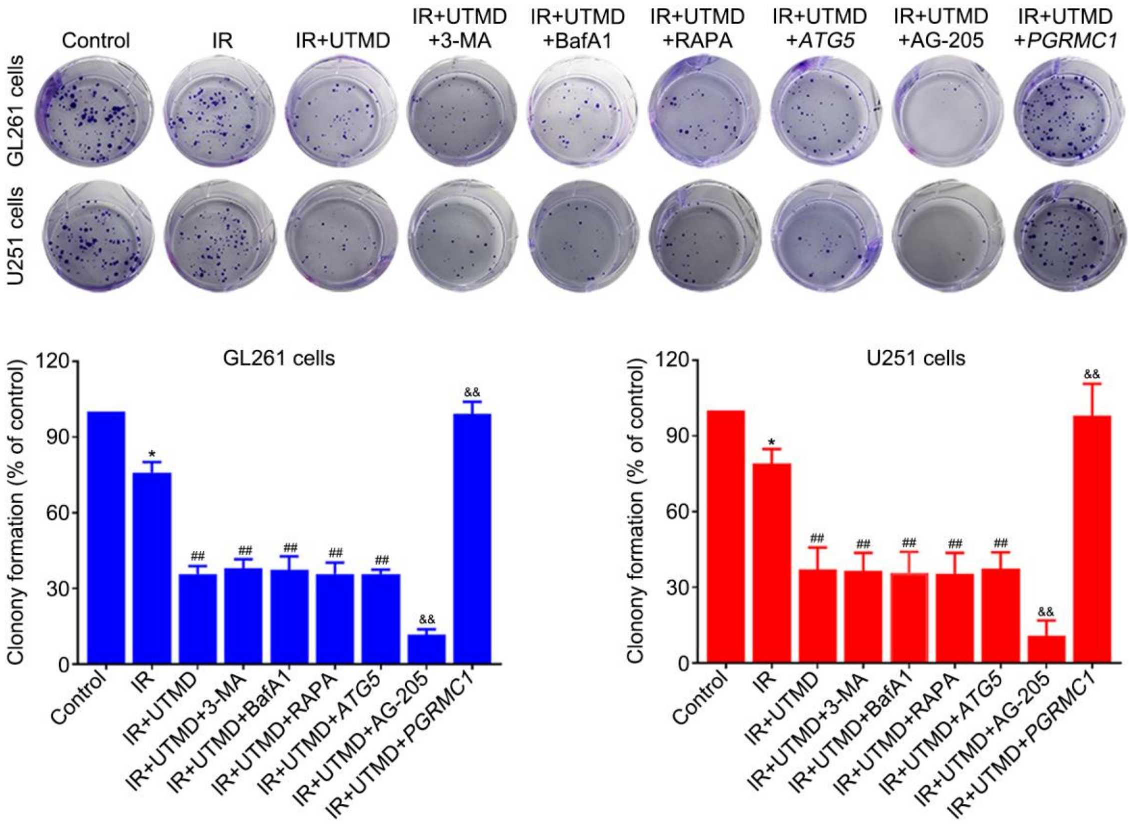

Fig. S2 Colony formation of glioblastoma cells was measured by clonogenic assay. GL261 and U251 cells were treated with 3-MA (5 mmol/L), BafA1 (10 nmol/L), AG-205 (10 μmol/L), or RAPA (20 nmol/L) for 1 h followed by IR (2 Gy) or IR plus UTMD treatment for another 24 h. Moreover, GL261 and U251 cells were transfected with lentiviral vectors encoding *AGT5* or *PGRMC1*. Then, cells were treated with UTMD followed by IR (2 Gy) exposure for another 24 h. The colony formation of glioblastoma cells was measured by clonogenic assay. Values are expressed as mean ± SD (n = 3). $^{*}P < 0.05$ vs. the vehicle-treated control group; $^{\#\#}P < 0.01$ vs. IR group; $^{\&\&}P < 0.01$ vs. IR plus UTMD group. IR ionizing radiation, UTMD ultrasound-triggered microbubble destruction, 3-MA 3-methyladenine, BafA1 bafilomycin A1, RAPA rapamycin, PGRMC1 progesterone receptor membrane component 1, SD standard deviation

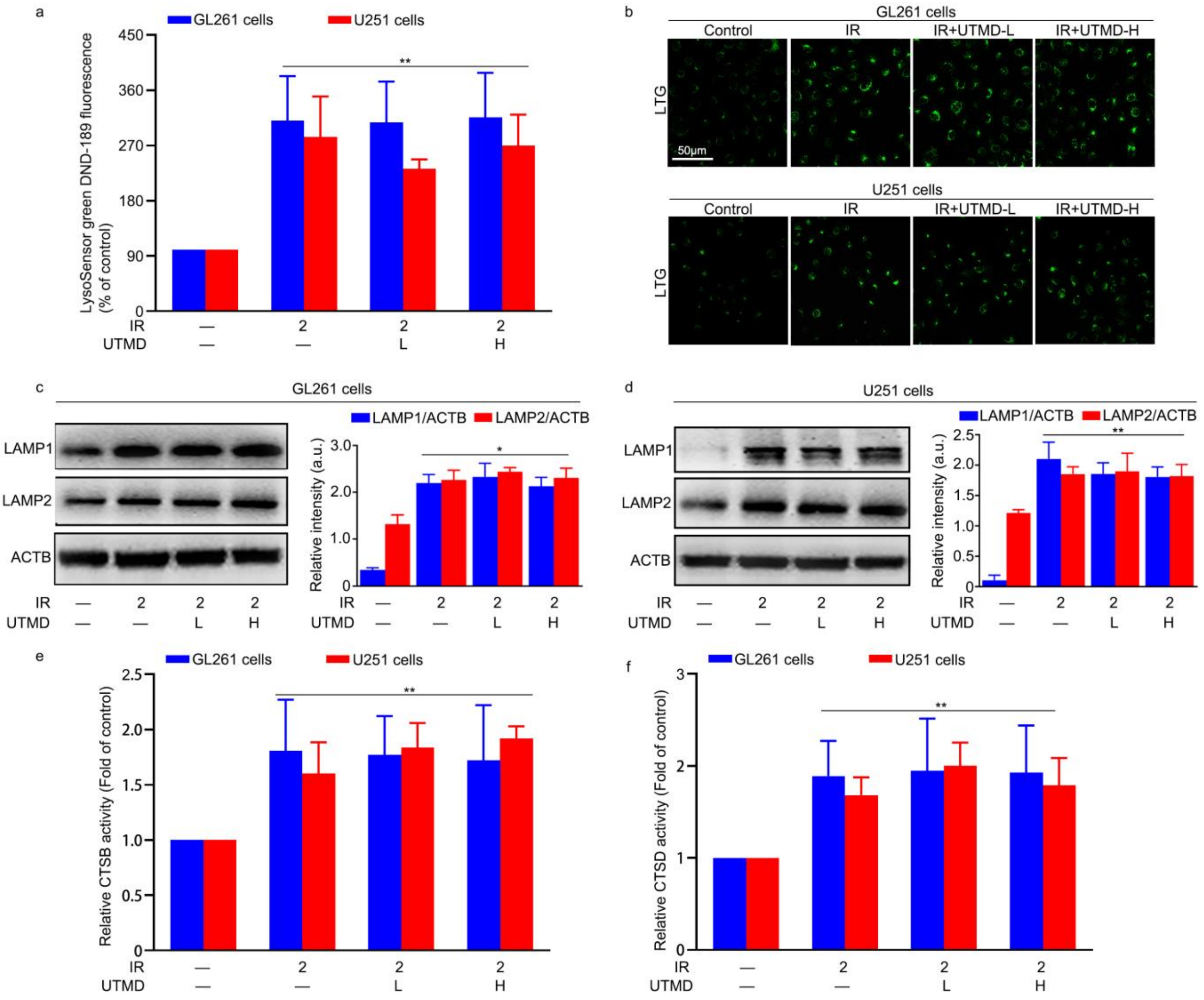

Fig. S3 UTMD had no effect on lysosomal function in IR-treated glioblastoma cells. GL261 and U251 cells were treated with UTMD-L (ultrasonic intensity of 1.2 W/cm^2 combined with 100 μl/ml MBs) or UTMD-H (ultrasonic intensity of 1.2 W/cm^2 combined with 200 μl/ml MBs) at a duty cycle of 10% for 60 s, and then the cells were exposed to IR (2 Gy). **a** LysoSensor DND-189 fluorescence intensity was quantified using an Infinite™ M200 Microplate Reader (Tecan Group Ltd.). **b** LTG fluorescence intensity was measured by a ZEISS LSM 900 confocal laser scanning microscope. **c-d** Expression of LAMP1, LAMP2 and ACTB was detected by Western blotting. The bar graph shows the quantification of the indicated proteins. **e-f** CTSB and CTSD activity was determined by a CTSB or CTSD activity kit, respectively. Values are expressed as mean ± SD (n = 3). $^{*}P < 0.05$, $^{**}P < 0.01$ vs. the vehicle-treated control group. IR ionizing radiation, UTMD ultrasound-triggered microbubble destruction, LTG LysoTracker green fluorescent dye, LAMP lysosomal-associated membrane protein, CTSB cathepsin B, CTSD cathepsin D, ACTB β-actin, MBs microbubbles, SD standard deviation, a.u. arbitrary units

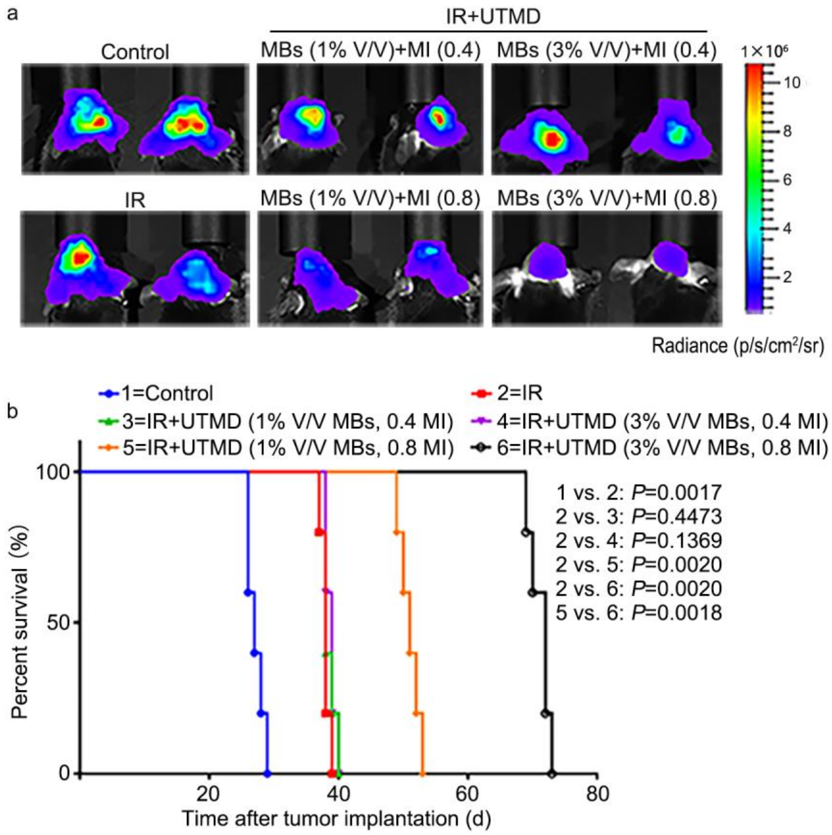

Fig. S4 Optimizing the parameters of UTMD to enhance the IR response of glioblastoma in vivo. Six-week-old female C57BL/6J mice were inoculated in the right caudate putamen with GL261 cells (1×10^5 cells per mouse) to establish the orthotopic glioblastoma mouse model. Seven days after transplantation, the tumor size was evaluated by in vivo bioluminescent imaging. Then, the animals were randomly allocated to treatment groups to receive control treatment or IR (everyday, 2 Gy/fraction, 5 fractions) combined with or without UTMD (every other day) treatment over a period of 5 d. On day 20 after tumor transplantation, bioluminescent imaging was used to determine the tumor size. **a** In vivo bioluminescent images (n = 6). **b** Kaplan-Meier survival analysis of orthotopic glioblastoma-bearing mice (n = 6). IR ionizing radiation, UTMD ultrasound-triggered microbubble destruction, MBs microbubbles, p/s/cm^2/sr photon per second per square centimetre per steradian
